# *Burkholderia cenocepacia* Prophages—Prevalence, Chromosome Location and Major Genes Involved

**DOI:** 10.3390/v10060297

**Published:** 2018-05-31

**Authors:** Bartosz Roszniowski, Siobhán McClean, Zuzanna Drulis-Kawa

**Affiliations:** 1 Institute of Genetics and Microbiology, University of Wroclaw, 51-148 Wroclaw, Poland; bartosz.roszniowski@gmail.com; 2 School of Biomolecular and Biomedical Science, University College Dublin, Belfield, Dublin 4, Ireland; siobhan.mcclean@ucd.ie

**Keywords:** *Burkholderia cenocepacia*, prophages, prevalence, in silico analyses

## Abstract

*Burkholderia cenocepacia*, is a Gram-negative opportunistic pathogen that belongs to *Burkholderia cepacia* complex (BCC) group. BCC representatives carry various pathogenicity factors and can infect humans and plants. Phages as bacterial viruses play a significant role in biodiversity and ecological balance in the environment. Specifically, horizontal gene transfer (HGT) and lysogenic conversion (temperate phages) influence microbial diversification and fitness. In this study, we describe the prevalence and gene content of prophages in 16 fully sequenced *B. cenocepacia* genomes stored in NCBI database. The analysis was conducted in silico by manual and automatic approaches. Sixty-three potential prophage regions were found and classified as intact, incomplete, questionable, and artifacts. The regions were investigated for the presence of known virulence factors, resulting in the location of sixteen potential pathogenicity mechanisms, including toxin–antitoxin systems (TA), Major Facilitator Superfamily (MFS) transporters and responsible for drug resistance. Investigation of the region’s closest neighborhood highlighted three groups of genes with the highest occurrence—tRNA-Arg, dehydrogenase family proteins, and ABC transporter substrate-binding proteins. Searches for antiphage systems such as BacteRiophage EXclusion (BREX) and Clustered Regularly Interspaced Short Palindromic Repeats (CRISPR) in the analyzed strains suggested 10 sequence sets of CRISPR elements. Our results suggest that intact *B. cenocepacia* prophages may provide an evolutionary advantage to the bacterium, while domesticated prophages may help to maintain important genes.

## 1. Introduction

The *Burkholderia* genus, named after its discoverer, Walter Burkholder [1,2] includes Gram-negative bacteria that cause plant disease (e.g., sour skin rot disease), but paradoxically they also act as natural pest antagonists by promoting plant growth [3,4]. There are also reports that *Burkholderia* lipopolysaccharide (LPS) is a factor which induces plant defensive capacity [5].

*Burkholderia cenocepacia* is a multidrug resistant, opportunistic pathogen. *B. cenocepacia* belongs to the *Burkholderia cepacia* complex (BCC), a group of 21 species of opportunistic pathogens that colonizes patients with cystic fibrosis (CF) and chronic granulomatous disease (CGD) [6]. Although, BCC representatives are responsible for only 3.5% of infections in CF patients, the fatality rate is higher than in the more commonly acquired *Pseudomonas aeruginosa* infection [7].

Following colonization of the host by BCC, the likelihood of complete eradication is very low. The course of infection differs depending on the CF status and may range from asymptomatic to severe inflammation of the lower respiratory tract, leading to lung function decline and ultimately to the patient’s death. Twenty percent of infected individuals develop “cepacia syndrome”, characterized by a high fever, pneumonia and severe bacteraemia [8]. Treatment is hindered by the pathogen’s inherent antimicrobial drug resistance, including insensitivity to fluoroquinolones, aminoglycosides and β-lactams [9]. Further, BCC bacteria are highly resistant to antimicrobial peptides and even has the ability to use penicillin G as a carbon source. BCC may form biofilms, further enhancing their resistance to antibiotics [10,11].

The mechanisms of BCC pathogenicity are multifactorial and complex. Because of that, finding solid strategies to combat infections caused by these bacteria is very challenging [12]. There are several known virulence mechanisms that have been reported to date: (i) invasion of epithelial cells and access to the bloodstream; (ii) structures responsible for adhesion (pili, flagella); (iii) LPS specific to Gram-negative bacteria; (iv) secretion systems (types I–IV); (v) biofilm formation; (vi) toxin-antitoxin (TA) systems; (vii) quorum sensing (QS) systems regulating expression of multiple virulence factors (e.g., toxins, proteases, lipases); and (viii) drug-resistance [13,14,15,16,17,18,19,20]. Of all the listed BCC virulence factors, only TA systems were described previously as encoded by prophages [21].

The genetic material of prophages is replicated by bacteria during its division, resulting in mutual benefits for the virus and its host. In addition, prophages can contribute to the enhancement of environment survival of infected cells by transferring genes which allow the exploitation of new nutrient sources and encode specific enzymes and toxins. Further, bacteriophages play a key role in the diversification of bacteria by immunizing their hosts against homologous viruses. All these factors can influence the survival of strains and changes in the composition of populations prevailing in a given environment. The process of gene donation by phages to the hosts is defined as lysogenic conversion [22]. It is suspected that prophages equip bacteria with toxins and enzymes, allowing their hosts to invade higher organisms and avoid the immune response and ultimately leading to increased pathogenicity of the bacteria [23]. Lysogenic conversion also plays an important role in transmitting antibiotic resistance. Such phenomenon was described for *Streptococcus*-specific phages transferring drug-resistance to chloramphenicol, macrolides, lincomycin and clindamycin [24].

Prophages can comprise a significant component of bacterial genetic material. In *Escherichia coli* O157 and *Streptococcus pyogenes* M3 MGAS 315, prophages account for up to 12% of the chromosomal DNA. In bacteria with smaller genomes, such as *Borrelia burgdorferi*, prophages exist as linear or circular episomes (plasmids). *B. burgdorferi* B31 is a particular example of such pseudolysogeny, as the episomal temperate phages may make up to 20% of its total genome [25]. It was assumed that this is caused by the evolutionary pressure of bacteria, which manifests in the removal of unessential DNA from its chromosome, maintaining the genome size at a particularly reduced level [26]. Bacteria hosting certain prophage may conduct a bacteriophage “domestication” process. This phenomenon is based on the modification of genetic material present in the cell, including nonsynonymous substitutions of nucleotides or deletions of whole genes (or regions). This leads to a partial or total disorder of the phage life cycle inhibiting prophage induction and lytic form of propagation. Overall, “domestication” results in the progressive removal of prophage genetic material, leaving only those sequences which are essential for bacteria. In most cases, these entities are referred to as cryptic phages. Once the phage DNA is just limited to individual genes or incomplete regions, it is referred to as a bacteriophage artifact [25]. The remaining phage regions may undergo further recombination with other prophages, plasmids and gene clusters which were already present within bacterial genome [27,28].

To date, there are 39 sequenced BCC phages in the NCBI database and two additional phage, phiK96243 and ϕH111-1, not in the database (Table S1). Phage phiK96243 and ϕH111-1 were described but their sequences were not submitted to any database as standalone sequence [21,29]. Twenty-three phages possess either integrase or recombinase (suggesting their temperate lifestyle) however only fourteen were labeled as temperate in the literature. Six of remaining phages are lytic, while 19 are unclassified. The taxonomical division places 19 phages in *Myoviridae*, 10 in *Podoviridae* and nine in *Siphoviridae*. The phiK96243 has not yet been classified.

In this study we analyzed 16 *B. cenocepacia* strains with fully sequenced genomes stored in the NCBI database. The main goals were to search for the presence and prevalence of prophages and their gene composition. Further, phage accumulation in particular chromosomes, location site (integration point), and closest neighborhood (flanking sequences) were also investigated. The occurrence of known virulence factors of *B. cenocepacia* was examined to determine if they were any of phage origin. All identified prophages were compared phylogenetically, to establish their taxonomic relationship. The analysis was done by in silico methods, based on two different approaches, one fully automatic and one manual approach to highlight the advantages and disadvantages of both techniques.

## 2. Materials and Methods

### 2.1. PHASTER Automated Annotation

Genomes of *B. cenocepacia* used for the analysis were obtained from the NCBI database (http://www.ncbi.nlm.nih.gov/) and were chosen based on completeness of sequence. Other incomplete sequences were omitted (as of 31.05.2017), leaving 16 bacterial genomes for further analysis (Table 1). Most *B. cenocepacia* strains have three chromosomes and each one was analyzed separately [30].

The prophages were located using PHASTER [31]. PHASTER scores potential phage regions using one of three methods, all of which are based on comparisons with the NCBI complete virus genome table (direct link to the table in citation) [32]. Based on the score, the region was assigned to one of three categories: intact (score higher than 90), questionable (score between 70 and 90) and incomplete (score below 70). The first method of scoring regions utilizes global comparison of the region with each of phages in the NCBI table as follows: if there is 100% or more conformity between total number of the CDS in both sources, the region score is set to 150. If the first method fails, the second or third one is used sequentially. The second evaluation is divided for three steps: (i) designation of the most probable potential phage (in the same manner as the first method, although with a minimum conformity level of 50%); (ii) the percentage comparison of the amount of proteins in the region and most probable potential phage, checked and multiplied by 100 (giving a partial score); (iii) the percentage comparison of the region and major potential phage length is checked and multiplied by 50 and added to the partial score from the step “ii”, giving the final score. The last method relies on the keywords contained in the names of phage specific proteins (also called “cornerstones”) present within particular region. PHASTER searches for words such as: “capsid”, “coat”, “fiber”, “head”, “integrase”, “lysin”, “plate”, “tail”, “portal”, “terminase”, “transposase”, etc. Each of those hits grants 10 points for particular region. Additionally, up to 20 points can be awarded, if the region size is greater than 30 kb and there are at least 40 proteins in it.

PHASTER runs the second and third method in parallel, leaving the higher score as the final one for particular region [31,33].

### 2.2. Manual Annotation

The annotation of prophage genomes was performed twice—once via a PHASTER automated annotation during the prophage region search and secondly by a manual search. During the manual annotation the open reading frames were delimited with Artemis (open reading frame (ORF) size equal or higher than 75 nt) and confirmed with GenMarkS [34,35]. All sequences of identified open reading frames were compared with the NCBI database using BLASTN and BLASTP and annotated accordingly [36,37]. In certain cases where BLAST results were questionable or were difficult to assess (hypothetical proteins), HMMER software was used to verify the protein structure and domains [38].

Manual annotation was enriched by additional verification of 10 open reading frames lying upstream and downstream of regions found by PHASTER, to confirm or refute their involvement in a potential prophage genome. The regions were searched for the presence of regulatory sequences during the manual annotation. The transfer RNA’s localization was conducted using Aragorn and confirmed by tRNAscan-SE [39,40]. Potential Rho-independent terminators were found using ARNold and curated manually [41]. The search for putative promoters was done by extraction of 100-nt sequences lying upstream of the predicted ORFs using STORM and their analysis by MEME [42,43]. MEME identified repetitive motifs which were investigated manually. Once identified, the sequence and localization of any regulatory sequences were marked in region characteristics cards (Supplementary Materials). Both annotation methods were compared in the tables (Tables S2–S17). Each table includes the manual and automatic annotation of one region, tabulating the localization of each ORF, the virus to which it holds homology as well as its accession numbers.

### 2.3. Phylogeny Analysis

Phylogeny reconstruction trees based on of the maximum likelihood method were prepared using Mega7. This software was set to use Jones–Taylor–Thornton (JTT) matrix-based model, while Neighbor–Join and BioNJ algorithms were applied to create an initial tree for heuristic search [44,45].

The graphical mapping of genomes was based on the PHASTER output data and confirmed by EasyFig 2.1 [46]. The location of regions in the host genomes was prepared with Circos (v. 0.67, Canada’s Michael Smith Genome Sciences Centre) [47].

## 3. Results

In the 16 *B. cenocepacia* genomes analyzed, sixty-three potential prophage sequences were detected (Table 2). Each region name was created based on its bacterial host with the addition of the chromosome number in which the prophage lay. The location of prophages and their initial completeness analysis were performed automatically with PHASTER to make three different categories: intact (marked in table as green), incomplete (marked red) and questionable (marked blue) (Table 2). Only intact regions were further annotated manually and compared in terms of taxonomy to known phages (based on NCBI database). The visualization of their genome map, investigation of regional neighborhood genes and phylogenetic comparisons were also done. The latter two analyses were also used for incomplete regions.

After initial automatic analysis of the qualified regions, detailed annotation of intact regions was conducted via Artemis and manual search with BLASTP. In addition, the data which indicated the presence of phage-borne DNA such as: (i) the presence of regions with higher G + C pairs; (ii) the presence of phage recombinase gene—integrase; (iii) the presence of pathogenicity factors of viral origin; (iv) the presence of the cornerstones, were also included into the study [48,49]. The cornerstones are highly conserved phage specific proteins, which serve as indicators of the presence of a potential prophage: large terminase subunit, portal protein, head maturation protease, coat protein, tail shaft protein, tail fiber protein and tail tape measure protein. Especially distinctive are groups of large terminase subunits and portal proteins as conservative viral proteins [25].

Taking into consideration all aforementioned conditions, it has been showed that PHASTER annotation often differed substantially from manual result. Three main characteristics have been noted for PHASTER regions analysis: (i) it often classifies genes as viral, even if its homology to the database was minimal (less than 25%); (ii) the gene sets in some regions either consist of multiple repeats of one gene or lack sufficient genes crucial for phage functioning; (iii) in contrast to the manual analysis, viral proteins were classified as phages specific to various bacterial taxonomical groups other than Burkholderia. Nonviral gene occurrence, gene repetitions and incorrect taxonomical affiliation of the genes were observed in the case of J2315_chr2_1, H111_chr1_3, 895_chr1_2, 895_chr1_3 and MSMB384WGS_chr1_2 regions which PHASTER marked as intact phages. Consequently, an additional group named by us as an artifact region (yellow) has been created. The artifact region group assembled all regions with homology less than 25% to the database or with gene set suggesting phage remnants.

In the case of two *B. cenocepacia* strains, J2315 and H111, a total of three phage regions have been already described in the literature. In *B. cenocepacia* J2315, chromosome 1, two prophages—KS10 (Goudie et al.) and Bcep-Mu (Summer et al.) were reported [50,51]. The third presided in the *B. cenocepacia* H111, chromosome 1—ϕH111-1 (Lynch et al.) [29]. Even though the ϕH111-1 was characterized thoroughly, its analysis was done based on the incomplete sequence of *B. cenocepacia* H111 (contigs). Because of that, the location of the ϕH111-1 was suggested on the second chromosome. As our study was conducted on the complete sequence of *B. cenocepacia* H111, the location of the ϕH111-1 could be indicated more precisely and was located on the first chromosome. [29]. Due to extensive characterization of regions containing KS10, BcepMu and ϕH111-1 in the literature, they were treated as intact phages and included only in phylogenetic analysis.

The final count of prophages, based on both automated and manual methods, revealed fifteen intact, nine questionable and thirty-four incomplete phages (Table 2). Furthermore, five regions were classified as artifacts—J2315_chr2_1, H111_chr1_3, 895_chr1_2, 895_chr1_3 and MSMB384WGS_chr1_2. Eight intact regions showed homology to Myoviridae (region homology ranged between 10.4% and 79.9%), four were similar to Siphoviridae (homology between 1.7% and 54.3%). The remaining three phages, KS10 and Bcep-Mu are described as Myoviridae, while ϕH111-1 belongs to Siphoviridae (Table S18). No correlation between host origin (CF or environmental) and number of phages have been detected.

Due to the observed differences between manual and automated annotation, the data obtained by both processes were compared in Tables S1–S16. PHASTER annotation occasionally did not indicate the gene function—only the gene number, such as gp12. To make the comparison table more consistent, a manual annotation was done for genes lacking a name, thus the basic information of the coding sequence was provided. This was done by a manual search of the databases for proteins with 100% homology or investigation of information stored in the NCBI database.

The interpretation of BLASTp results and occurrence of ORFs differed locally between manual and automatic annotation in all of analyzed genomes. Variations in genome start positions were noted for regions: 895_chr1_9, FL-5-3-30-S1-D7_chr1_2 and J2315_chr1_1. Furthermore, the latter phage also had an altered genome end position.

In most of the potential prophage genomes (except artifact regions) the differences in annotation were not sufficiently significant to change the final classification of prophage completeness. However, in the case of J2315_chr2_1, H111_chr1_3, 895_chr1_2, 895_chr1_3 and MSMB384WGS_chr1_2 the result of the manual annotation led to a change of status from an intact to an artifact region.

Supplementary data (Tables S1–S16) were used to create the comparative maps (Figures S1–S16) depicting the constitution of each prophage genome. Figure 1 presents the comparative map of J2315_chr1_1 phage region, while the remaining maps are shown in Supplementary Materials (Figures S1–S16). Each image shows two variants of phage genome annotation and approximate location of prophage in the host chromosome. Most crucial genes with determined function are colored likewise on both annotation versions to provide further simplification of compared methods. Based on the results gathered in the Table 2, the genome maps depicting the location of identified regions was created with Circos (Figure 2).

The intact regions were compared with the BCC phages available in the NCBI database and with each other (Table S18). The inquiry against the database returned following results: (i) phages J2315_chr1_1 (41.28%), 895_chr1_7 (33.54%) and CR 318_chr1_1 (74.62%) showed high similarity to phage KL3 (NC_015266.1); (ii) phages VC7848_chr1_2 (78.02%) and MC0-3_chr1_1 (63.70%) showed high similarity to phage AP3 (KP966108.1); (iii) phages DWS 37E-2_chr1(1.70%) and FL-5-3-30-S1-D7_chr1_2 (1.70%) showed minimal similarity to phage phi1026b (NC_005284.1); (iv) phage 895_chr1_9 showed 54.29% similarity to KS9 (NC_013055.1); (v) phage 895_chr2_1 showed 61.17% similarity to ST79 (NC_021343.1); (vi) phage DDS 22E-1_chr1_2 showed 21.50% similarity to phi644 (NC_009235.2); (vii) phage VC12308_chr1_1 showed 46.44% similarity to phi52237 (NC_007145.2); while (vii)—phage 895_chr1_1 showed 79.90% similarity to BcepMu (NC_005882.1). The comparison among studied phages showed a high similarity between phages J2315_chr1_1, VC12308_chr1_1 and 895_chr1_7. Another similar phages were MC0-3_chr1_1 and VC7848_chr1_1. The similarity between the other phages examined was not significant (Table S18).

Potential bacteriophages found in the bacterial genomes differed in the prevalence and distribution within host chromosomes. To investigate if there was any particular pattern in the placement of prophages in *B. cenocepacia* strains, the amount and location of prophage regions were examined. Table 3 presents the total prevalence of phage genomes in *B. cenocepacia* chromosomes. The occurrence of phages in hosts genomes varied from 0.3% to 3.67% of total DNA content, while the virus abundance in a single chromosome was between 0.3% up to 4.29%. Regardless of the number of chromosomes in a particular strain, the majority of potential phage regions were always found in chromosome 1. In 11 out of 12 strains possessing three chromosomes, most of prophage sequences were divided between chromosome 1 and 3, with a dominance of chromosome 1 (seven out of eleven). In the case of two-chromosome strains the ratio of phage to host sequence did not have any distinctive division.

To investigate prophage integration sites in the host chromosomes we manually examined their adjacent regions in the adjacent neighborhood. This verification was done regardless of the region status (intact, incomplete, questionable, artifact) (Table S18). Two approaches were used. First, we determined single genes located next to start and end positions. Second, we considered the 5–10 kb region located next to phage genome start and end. The first study aimed to search for a pattern in integration site (similar neighboring gene). The second method sought to verify the positioning of the start and end positions which were determined automatically by PHASTER. If the verification showed the presence of additional phage-like genes, the appropriate change was done in manual version of annotation. In most cases the phage genomes started and ended in the intergenic space. There were however several exceptions: (i) start position of prophage regions located inside the host gene were observed for J2315_chr1_2, 895_chr1_3, 895_chr1_4, HI2424_chr2_1, HI2424_chr3_1, DDS 22E-1_chr2_1; (ii) end position of prophage regions located inside the host gene were observed for DWS 37E-2_chr1_1, 895_chr1_3, 895_chr1_8 and VC12308_chr1_1; (iii) both, start and end positions of prophage regions located inside the host gene were observed for 895_chr1_3 (artifact region).

The closest neighbors of phage regions were often hypothetical proteins, which flanked phage genomes in 48 cases (37.2%). The most common neighboring, recognizable sequence (non-hypothetical protein), was tRNA-Arg, which was found either upstream or downstream of the prophage sequence (10.3% of all regions). The second group of sequences located next to identified regions was ABC transporter genes (4.7% of all regions), while the third one was LysR family transcriptional regulator genes (4%). Peptidase encoding genes were found in the neighborhood of three phage regions (2.4%). The remaining 52 flanking genes (41.4%) have determined function, however they did not repetitively appear as neighboring sequences (Table S18).

Bacteriophages can encode virulence factors such as toxins, enzymes or drug resistance. As bacteria may domesticate phages by leaving only useful genes, all identified prophage regions (complete, questionable, artifact, incomplete) were analyzed for the presence of potential virulence genes (Table 4). Thirteen genes were recognized as sequences potentially increasing bacterial drug resistance: (i) one was specified as class A β-lactamase; (ii) one was the Vicinal Oxygen Chelate (VOC) family protein; (iii) eleven belonged to the Major Facilitator Superfamily (MFS) transporter superfamily. VOC proteins possess Glo_EDI_BRP_like domains, which are found in a vast variety of related groups of metalloproteins. One of these groups comprised of antibiotic resistance proteins which can block drugs in different ways. For example, bleomycin resistance protein inhibits drug activity by binding to it, while fosfomycin resistance proteins inactive the drug by modifying its molecule [52,53].

**Figure 3:**
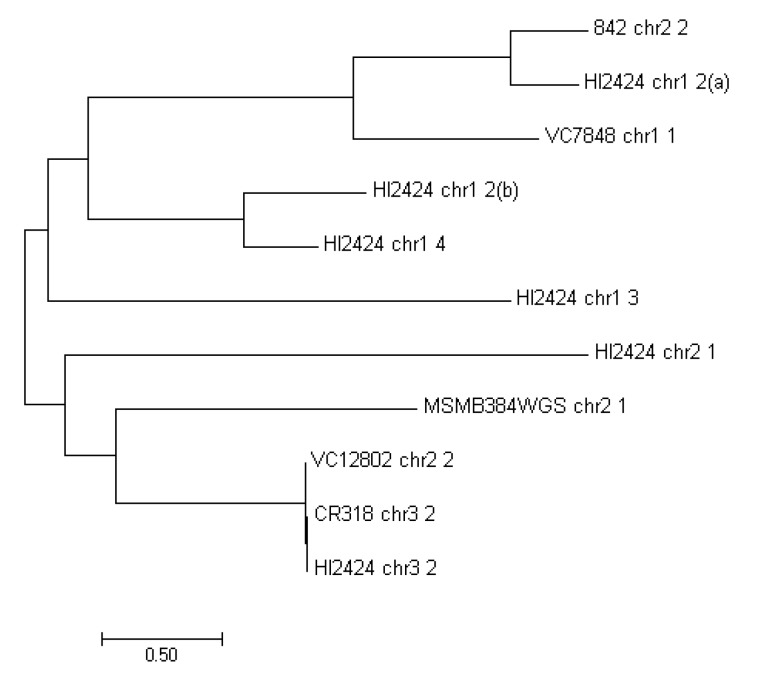
Maximum likelihood comparison of the MFS proteins found in examined strains.

The MFS transporters are the largest known superfamily of secondary carriers [54]. A considerable amount of drug and multidrug efflux pumps found in bacteria are comprised of the MFS transporters class. They are found in both Gram-negative (e.g., enterobacteria, *Pseudomonas* sp. or *Moraxella* sp.) and Gram-positive strains (*Staphylococcus* sp., *Bacillus* sp.), however it is unclear if they share substrate profiles [55]. The MFS found in strains examined have been phylogenetically compared to each other (Figure 3). Although, MFS proteins occur six times in the analyzed regions of HI2424, they differ significantly. There is however high similarity between MFS transporters found in VC12802_chr2_2, CR318_chr3_2 and HI2424_chr3_2 (undelined in Table 4).

Three genes encoded for toxin–antitoxin (TA) systems (Table 4). The hicA and hicB found in DDS 22E-1_chr1_2 encode a complete two protein system that targets mRNA. HicA is the predicted interferase, while HicB balances the system and neutralizes HicA [56]. Interestingly, the only complete TA system was found in intact phage DDS 22E-1_chr1_2. This may suggest that the host is not able to remove or damage the phage because the system is active.

Bacteria can be armed with antiphage defense systems such as CRISPR-Cas and BREX, which effectively protect its host against viral infections [57]. Those systems may also take part in disposing of integrated phages (completely or partially), leading to the creation of cryptic phages [58]. Consequently, all bacterial genomes were screened for the presence of these bacterial antiphage defense systems. No BREX systems were found after manual analysis based on previously described methodology [59]. In contrast, using CRISPRfinder [60] ten potential CRISPR sequences were located in the chromosomes of *B. cenocepacia* strain 842 (two sequences in the same chromosome), J2315, H111, DWS 37E-2, 895, ST32, MSMB384WGS, CR318, DDS 22E-1, and VC7848 strains. Table S19 presents detailed data considering each of potential CRISPR sequence (Supplementary Data).

**Figure 4:**
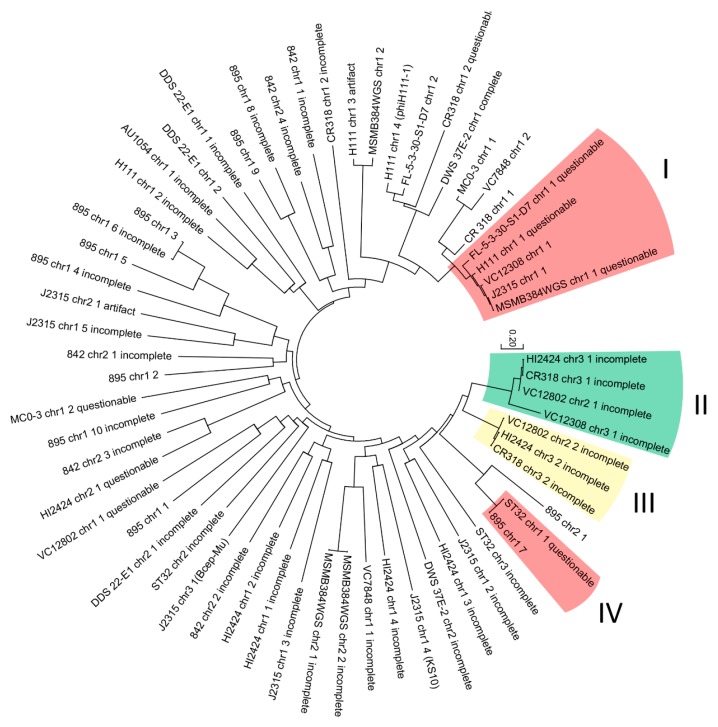
Maximum likelihood comparison of 64 nucleotide phage-like sequences found in *B. cenocepacia* strains.

In an attempt to differentiate taxonomical groups of analyzed prophages, a phylogenetic analysis was conducted. The evolutionary resemblance of 64 nucleotide phage-like sequences (intact, artifact, questionable, incomplete) were proposed by using the maximum likelihood method based on the Tamura-Nei model [61]. The tree with the highest log likelihood (−108,132.8877) is presented in Figure 4. The tree is drawn to scale, with branch lengths measured in the number of substitutions per site.

Four specific clades were distinguished. Phage genomes located in selected clades (marked on the tree) share the same neighboring sequence in the host genome: clade I and IV—tRNA-Arg; clade II—dehydrogenase family proteins; clade III—ABC transporter substrate-binding proteins. Other branches did not show any significant internal resemblance.

To summarize the results obtained by automatic and manual methods, region characteristics cards were created for all intact and artifact regions (Supplementary data). The cards collect the most crucial data relating to each of the qualified phages. The intact phage card consists of basic data including size and location, taxonomical affiliation (based on the homology the NCBI database), the number of ORFs annotated, recognized regulatory sequences, region derivation and the complete annotation. Information regarding regulatory sequences and taxonomy are not included in artifact region cards however the additional data including homology of each protein is appended to the annotation tables.

## 4. Discussion

This article presents the results of an in silico analysis of potential prophage regions found in *Burkholderia cenocepacia* genomes, currently stored in the NCBI database (October 2017). Regions were divided into four groups basing on their completeness: intact (15 regions), questionable (nine regions), incomplete (34 regions) and artifact (five regions). Phage prevalence differed between BCC strains but did not exceed 3.67% of the total genome size, which is relatively low ratio in comparison to reports by Ohnishi et al. and Beres et al. considering different bacterial genus, such as *E. coli* O157 or M3 MGAS 315 (12% of the chromosome) [62,63].

All regions were subjected to both automatic and manual annotation processes to see if there were notable differences in these techniques. The automatic method was fast and allowed rough annotations, but it was quite imprecise because of incorrect interpretation of the homology level between genes in regions and in the database. Even if the genes’ similarity to the virus database was at an insignificant level (in some cases as low as 5%) the software identified the gene as viral. In the PHASTER scoring method some regions consisting of multiple repeats of the same virus gene were eventually marked as intact prophages. Bearing that in mind, the automatic annotation was verified manually, resulting in localization of potential mistakes made by the software and allowing the creation of an additional group of artifact regions. Those regions were either incomplete prophages with essential genes deprived or were aggregates of random viral-origin genes (often repetitive). The occurrence of such regions may suggest that BCC strains are able to domesticate prophages, as described previously by Bobay et al. [27].

Recently, Bodilis et al. 2018 [63] compared the *Burkholderia* ET12 lineage with a selection of environmental strains, including similar analysis based on the PHASTER software. These results considering fully sequences strains (J2315, MC0-3 and AU1054) presented by Bodilis et al. 2018 [63] were partially synonymous with the ones obtained in this study: (i) J2315_chr1_1 is the same region as the PI_J2315_1; (ii) J2315_chr2_1 is the same region as the PI_J2315_3; (iii) both prophages which were already described (KS10 and BcepMu) have been annotated in the same regions by all studies [50,51]; (iv) regions MC0-3_chr1_1 and MC0-3_chr1_2 are the same regions respectively to PI_MC0-3_1 and PI_MC0-3_2. Regions PI_J2315_6, PI_MC0-3_3, PI_MC0-3_4 and PI_AU1054_1 presented by Bodilis et al. 2018 [63] could not be found with the current version of PHASTER, while region PI_J2315_4 (ORFs BCAM0001AM_1973–BCAM0001AM_2038) is no longer available in the database, making it impossible to address those regions in the comparison. Further, all data relating to the H111 genome were based on the contigs sequence and therefore, the PI_H111_1, PI_H111_2 and PI_H111_3 regions could not be located in current database. Additionally, the size of the region PI_H111_3 which has been described as phage ϕH111-1 does not match the region size from our study and Lynch et al. description (10.8 kB vs. 43.0 kB) [29,64].

A search for virulence factors was conducted in all regions to examine eventual influence of prophage to host pathogenicity. The results obtained suggest that prophages of *Burkholderia* preferentially carry drug resistance mechanisms over other categories of virulence factors. This finding is consistent to those of Summer et al. and Ronning et al. [21,65].

The resistance of BCC to β-lactams is well-known, however there are no reports on these genes being located on the prophage region [62]. We now show that a class A β-lactamase encoding gene was identified in 842_chr2_1 region. MFS transporter genes were the most commonly observed in BCC genomes analyzed which is probably related to host drug resistance. MFS have a very broad substrate spectrum, thus their influence on the host virulence could not be precisely indicated. DeShazer examined the influence of MFC genes deletions in *B. mallei* prophage phi1026b extensively, however no correlation to the host virulence was observed [66].

Three of the identified genes—fic (J2315_chr2_1), hicA and hicB (DDS 22E-1_chr1_2), encode components of toxin–antitoxin systems. Fic is a representative of incomplete Fic/Doc system, whereas hicA and hicB form a complete hicAB cassette. The HicAB cassette has been extensively described and classified as a type II toxin-antitoxin system [67]. HicA serves as a ribosome-independent mRNA interferase, which can cleave specific mRNAs and tmRNAs. HicB function as antitoxin and is composed of two domains, a DNA-recognition domain (C-terminal) and an RNase H fold with no catalytic residues (N-terminal). The HicAB cassette was previously identified as a part of bacteria (including *Burkholderia* species) or prophage genomes [56,68]. What is more, the HicA protein, found in *Burkholderia pseudomallei*, was proven to play a role in formation of the persister cells tolerant to ciprofloxacin and ceftazidime [69]. There are no reports of locating a HicAB cassette directly in a *Burkholderia* prophage.

The virulence of *B. cenocepacia* strains HI2424, AU1054 and J2315 was tested in the nematode *Caenorhabditis elegans* model by Cooper et al. 2009 [69]. It was showed that two closely related *B. cenocepacia* isolates of the PHDC clonal lineage, HI2424 and AU1054, exhibited significantly different virulence, with HI2424 being more lethal and toxic to the nematodes. When considered in relation to our study, it could be inferred that strains possessing more prophage regions are more virulent/toxic [70].

The results obtained from the CRISPR system search did not show any direct correlation between the host and its prophages. The number of phages identified and their completeness differed between hosts possessing CRISPR system. The 65% of the CRISPR systems found were located on the second chromosome, which was the least favored prophage integration place (based on the prophage prevalence and distribution). If the strain carried CRISPR on the second chromosome there were no prophages present or only incomplete ones. The results indicating the presence of CRISPR differed from the study of Bodilis et al. 2018 [64]. While the absence of CRISPR in H111 may be explained by the incomplete sequence of H111, the lack of the anti-phage system in J2315 is more difficult to interpret. The most probable reason is the use of a more recent version of the CRISPRfinder which is more effective in searching for these systems.

The closest neighborhood analysis showed that the biggest fraction of repetitive sequences found next to the prophage regions was tRNA-Arg. The tRNAs are known to be the most common integration sites of prophages, including BCC hosts. Previously described tRNA that are known to be valid recombination sites are tRNA-Arg (Roszniowski et al.), tRNA-Thr (Lynch et al.), tRNA-Phe (Ronning et al.) and tRNA-Ser (Holden et al.) [29,65,71,72]. Even though the ABC transporter, LysR family transcriptional regulators and peptidase encoding genes were found in the phage neighborhood less frequently than tRNA-Arg, their continual appearance, may suggest these are also plausible integration sites for viruses (Table S18).

In summary, the prophages identified in the *B. cenocepacia* genomes analyzed differed in amount, composition and completeness. The biggest proportion of located viruses were marked as incomplete, which may suggest that this host is actively domesticating its prophages. The fact that BCC are environmental bacteria associated with the rhizosphere also suggests that it will favor collection of phage genes, as they may promote the fitness in their habitat. The analyses allowed antiphage systems to be distinguished as well as phage genes, which are potentially responsible for host virulence (drug resistance, pathogenicity). The results obtained offer profound insights into the composition of each phage genome, which are future targets for research, and will enable the selection of appropriate methods and build a solid foundation for further experimental work in relatively cost-efficient and quick manner.

## Figures and Tables

**Figure 1:**
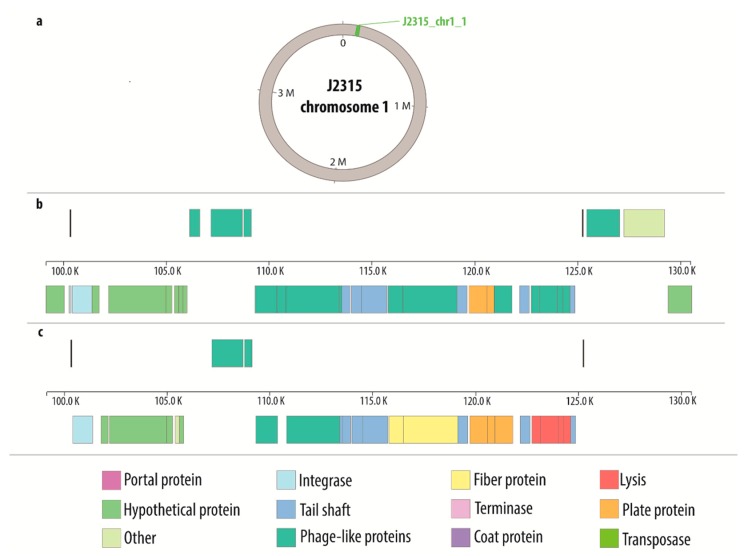
Comparative map of J2315_chr1_1 phage region: (**a**) general location of the phage on the host chromosome; (**b**) genes characterization based on PHASTER annotation; (**c**) genes characterization based on manual annotation.

**Figure 2:**
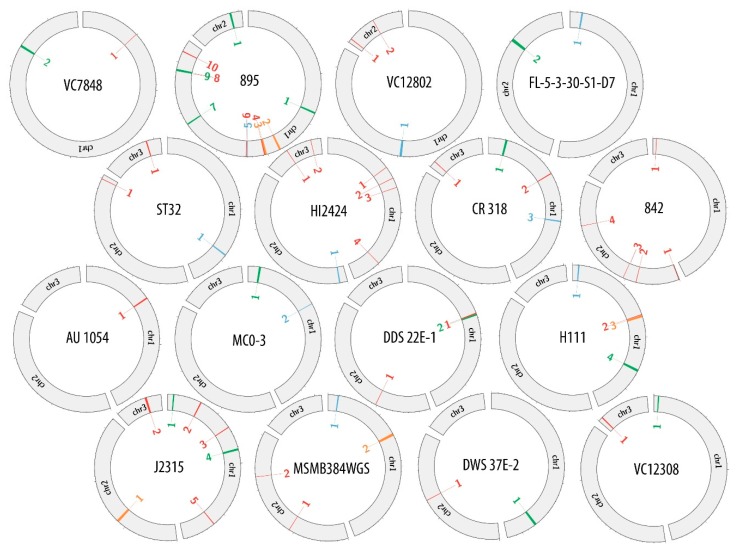
Genome maps of investigated *B. cenocepacia* strains depicting the location of found phage regions.

**Table 1:** *B. cenocepacia* strains chosen for analysis.

No.	Strain	Origin	Chromosome	A/N	Genome Size (bp)
1	*B. cenocepacia* J2315	CF	1	NC_011000.1	3,870,082
			2	NC_011001.1	3,217,062
			3	NC_011002.1	875,977
2	*B. cenocepacia* H111	CF	1	NZ_HG938370.1	3,572,953
			2	NZ_HG938371.1	3,102,677
			3	NZ_HG938372.1	1,039,263
3	*B. cenocepacia* DWS 37E-2	Soil	1	NZ_CP007781.1	3,241,886
			2	NZ_CP007780.1	2,375,865
			3	NZ_CP007779.1	994,670
4	*B. cenocepacia* FL-5-3-30-S1-D7	Soil	1	CP013397.1	3,461,321
			2	CP013396.1	2,869,430
5	*B. cenocepacia* 895	Sepsis (neonatal)/Cord blood	1	NZ_CP015036.1	7,459,003
			2	NZ_CP015037.1	1,072,666
6	*B. cenocepacia* ST32	CF	1	NZ_CP011917.1	3,822,749
			2	NZ_CP011918.1	3,086,109
			3	NZ_CP011919.1	989,585
7	*B. cenocepacia* 842	Nasal inflammation/non-CF	1	NZ_CP015033.1	3,526,250
			2	NZ_CP015034.1	3,107,451
			3	NZ_CP015035.1	1,271,875
8	*B. cenocepacia* MSMB384WGS	Water	1	NZ_CP013450.1	3,588,848
			2	NZ_CP013452.1	3,069,864
			3	NZ_CP013451.1	1,121,886
9	*B. cenocepacia* HI2424	Soil	1	NC_008542.1	3,483,902
			2	NC_008543.1	2,998,664
			3	NC_008544.1	1,055,417
10	*B. cenocepacia* CR318	Plant root	1	NZ_CP017238.1	3,511,146
			2	NZ_CP017239.1	3,097,552
			3	NZ_CP017240.1	1,056,196
11	*B. cenocepacia* AU 1054	CF	1	NC_008060.1	3,294,563
			2	NC_008061.1	2,788,459
			3	NC_008062.1	1,196,094
12	*B. cenocepacia* MC0-3	Soil	1	NC_010508.1	3,532,883
			2	NC_010515.1	3,213,911
			3	NC_010512.1	1,224,595
13	*B. cenocepacia* DDS 22E-1	Aerosol sample	1	NZ_CP007783.1	3,668,832
			2	NZ_CP007784.1	3,209,624
			3	NZ_CP007782.1	1,166,794
14	*B. cenocepacia* VC7848	CF	1	NZ_CP019668.1	7,499,459
15	*B. cenocepacia* VC12308	CF	1	NZ_CP019674.1	3,668,000
			2	NZ_CP019672.1	2,984,720
			3	NZ_CP019673.1	964,521
16	*B. cenocepacia* VC12802	CF	1	NZ_CP019670.1	6,339,862
			2	NZ_CP019669.1	1,055,047

CF—sample collected from cystic fibrosis patient.

**Table 2:** Presence of prophages in *Burkholderia cenocepacia* genomes stored in the NCBI database. Regions types were marked with colors: intact (green), questionable (blue) incomplete (red) and artifact region (yellow). Table containing more detailed data can be found in Supplementary data (Table S18).

Host Name	Chromosome	Region Name	Phage Genome Size (bp)	Status	Location in Host Genome	
					Start	End
J2315	1	J2315_chr1_1	24,997	intact	100,299	125,296
		J2315_chr1_2	22,458	incomplete	620,851	643,309
		J2315_chr1_3	16,209	incomplete	1,301,322	1,317,531
		J2315_chr1_4 (KS10)	37,369	intact	1,729,077	1,766,446
		J2315_chr1_5	15,015	incomplete	3,241,582	3,256,597
	2	J2315_chr2_1	46,822	artifact region	1,140,168	1,186,990
	3	J2315_chr3_1 (Bcep-Mu)	37,581	intact	572,009	609,590
H111	1	H111_chr1_1	22,907	questionable	100,107	123,014
		H111_chr1_2	10,399	incomplete	1,593,686	1,604,085
		H111_chr1_3	38,387	artifact region	1,609,303	1,647,690
		H111_chr1_4 (ϕH111-1)	43,024	intact	2,595,517	2,638,541
	2	-	-	-	-	-
	3	-	-	-	-	-
DWS 37E-2	1	DWS 37E-2_chr1	36,859	intact	2,747,954	2,784,813
	2	DWS 37E-2_chr2	8671	incomplete	1,323,368	1,332,039
	3	-	-	-	-	-
FL-5-3-30-S1-D7	1	FL-5-3-30-S1-D7_chr1_1	23,101	questionable	167,573	190,674
		FL-5-3-30-S1-D7_chr1_2	44,062	intact	2,017,976	2,062,038
	2	-	-	-	-	-
895	1	895_chr1_1	34,705	intact	2,755,051	2,789,756
		895_chr1_2	38,627	artifact region	3,737,465	3,776,092
		895_chr1_3	43,300	artifact region	4,023,532	4,066,832
		895_chr1_4	14,687	incomplete	4,061,645	4,076,332
		895_chr1_5	14,593	questionable	4,398,712	4,413,305
		895_chr1_6	14,946	incomplete	4,416,973	4,431,919
		895_chr1_7	26,430	intact	5,741,502	5,767,932
		895_chr1_8	29,290	incomplete	6,823,839	6,833,750
		895_chr1_9	40,988	intact	6,823,839	6,864,827
		895_chr1_10	18,325	incomplete	7,212,238	7,230,563
	2	895_chr2_1	37,652	intact	826,395	864,047
ST32	1	ST32_chr1	23,895	questionable	2,869,763	2,893,658
	2	ST32_chr2	10,694	incomplete	2,980,754	2,991,448
	3	ST32_chr3	20,608	incomplete	850,166	870,774
842	1	842_chr1_1	10,037	incomplete	57,007	67,044
	2	842_chr2_1	8621	incomplete	6423	15,044
		842_chr2_2	9739	incomplete	791,624	801,363
		842_chr2_3	7465	incomplete	1,038,705	1,046,170
		842_chr2_4	7448	incomplete	2,281,847	2,289,295
	3	-	-	-	-	-
MSMB384WGS	1	MSMB384WGS_chr1_1	24,156	questionable	172,483	195,500
		MSMB384WGS_chr1_2	48,964	artifact region	1,394,910	1,443,874
	2	MSMB384WGS_chr2_1	8810	incomplete	1,087,426	1,096,236
		MSMB384WGS_chr2_2	7306	incomplete	2,234,278	2,241,584
	3	-	-	-	-	-
HI2424	1	HI2424_chr1_1	8280	incomplete	1,165,247	1,173,527
		HI2424_chr1_2	9114	incomplete	1,383,513	1,392,627
		HI2424_chr1_3	8107	incomplete	1,556,642	1,564,749
		HI2424_chr1_4	7746	incomplete	2,976,934	2,984,680
	2	HI2424_chr2_1	20,628	questionable	126,942	147,570
	3	HI2424_chr3_1	8809	incomplete	421,282	430,091
		HI2424_chr3_2	8134	incomplete	872,989	881,123
CR 318	1	CR 318_chr1_1	38,476	intact	300,609	339,084
		CR 318_chr1_2	16,791	incomplete	1,309,949	1,326,739
		CR 318_chr1_3	22,450	questionable	2,180,663	2,203,112
	2	-	-	-	-	-
	3	CR 318_chr3_1	11,919	incomplete	112734	124652
		CR 318_chr3_2	7542	incomplete	570,308	577,849
AU 1054	1	AU 1054_chr1_1	24,119	incomplete	1,172,051	1,196,169
	2	-	*-*	*-*	*-*	*-*
	3	-	*-*	*-*	*-*	*-*
MC0-3	1	MC0-3_chr1_1	38,872	intact	198,439	237,310
		MC0-3_chr1_2	10,797	questionable	1,405,878	1,416,674
	2	-	*-*	*-*	*-*	*-*
	3	-	*-*	*-*	*-*	*-*
DDS 22E-1	1	DDS 22E-1_chr1_1	24,368	incomplete	1,606,100	1,630,467
		DDS 22E-1_chr1_2	31,311	intact	1,625,635	1,656,945
	2	DDS 22E-1_chr2_1	9602	incomplete	1,022,571	1,032,172
	3	-	*-*	*-*	*-*	*-*
VC7848	1	VC7848_chr1_1	7080	incomplete	983,606	990,685
		VC7848_chr1_2	38,294	intact	6,353,033	6,391,326
VC12308	1	VC12308_chr1_1	22,704	intact	70,339	93,042
	2	-	-	-	-	-
	3	VC12308_chr3_1	20,452	incomplete	73,079	93,531
VC12802	1	VC12802_chr1_1	39,072	questionable	3,934,787	3,973,858
	2	VC12802_chr2_1	11,921	incomplete	63,441	75,362
		VC12802_chr2_2	7545	incomplete	546,849	554,394

**Table 3:** The prevalence of prophages within *B. cenocepacia* genomes.

Host	Chromosome	Chromosome Size (bp)	Phage Prevalence in Chromosome (%)	Potential Phage Regions in Chromosome	Total Phage Prevalence in the Host Genome (%)
J2315	1	3,870,082	2.99	5	2.51
	2	3,217,062	1.45	1	
	3	875,977	4.29	1	
H111	1	3,572,953	3.24	4	1.50
	2	3,102,677	-	0	
	3	1,039,263	-	0	
DWS 37E-2	1	3,241,886	0.71	1	0.81
	2	2,375,865	0.36	1	
	3	994,670	-	0	
FL-5-3-30-S1-D7	1	3,461,321	1.94	2	1.06
	2	2,869,430	-	0	
895	1	7,459,003	3.70	10	3.67
	2	1,072,666	3.51	1	
ST32	1	3,822,749	0.67	1	0.69
	2	3,086,109	0.34	1	
	3	989,585	2.08	1	
842	1	3,526,250	0.28	1	0.54
	2	3,107,451	1.07	4	
	3	1,271,875	-	0	
MSMB384WGS	1	3,588,848	2.04	2	1.15
	2	3,069,864	0.52	2	
	3	1,121,886	-	0	
HI2424	1	3,483,902	0.95	4	0.93
	2	2,998,664	0.69	1	
	3	1,055,417	1.60	2	
CR 318	1	3,511,146	2.21	3	1.27
	2	3,097,552	-	0	
	3	1,056,196	1.84	3	
AU 1054	1	3,294,563	0.73	1	0.30
	2	2,788,459	-	0	
	3	1,196,094	-	0	
MC0-3	1	3,532,883	1.40	2	0.62
	2	3,213,911	-	0	
	3	1,224,595	-	0	
DDS 22E-1	1	3,668,832	1.52	2	0.80
	2	3,209,624	0.30	1	
	3	1,166,794	-	0	
VC7848	1	7,499,459	0.60	2	0.60
VC12308	1	3,668,000	0.62	1	0.56
	2	2,984,720	-	0	
	3	964,521	2.12	1	
VC12802	1	6,339,862	0.61	1	0.79
	2	1,055,047	1.84	2	

**Table 4:** Potential virulence factors located in found regions. Regions types were marked with colors: intact (green), questionable (blue), incomplete (red) and artifact region (yellow).

Region	Start	End	Product	Virulence Effect	Accession
J2315_chr2_1	12,317	12,700	Fic	TA system compound	WP_006488862.1
842_chr2_1	6642	7541	class A β-lactamase	drug resistance	WP_034202207.1
842_chr2_2	2065	3390	MFS transporter	drug resistance/virulence	WP_006495119.1
MSMB384WGS_chr2_1	1	1500	MFS transporter	drug resistance/virulence	WP_060268128.1
MSMB384WGS_chr2_1	2697	3080	VOC family protein	drug resistance/virulence	WP_060268132.1
HI2424_chr1_2	1	1311	MFS transporter * (a)	drug resistance/virulence	WP_011545048.1
HI2424_chr1_2	3764	4969	MFS transporter * (b)	drug resistance/virulence	WP_011545051.1
HI2424_chr1_3	1039	2301	MFS transporter	drug resistance/virulence	WP_011545193.1
HI2424_chr1_4	2829	4004	MFS transporter	drug resistance/virulence	WP_011694391.1
HI2424_chr2_1	3597	4892	MFS transporter	drug resistance/virulence	WP_011548498.1
HI2424_chr3_2	3269	4666	MFS transporter	drug resistance/virulence	WP_011695034.1
CR 318_chr3_2	1499	2896	MFS transporter	drug resistance/virulence	WP_011695034.1
DDS 22E-1_chr1_2	9157	9552	HicB	TA system compound	AJT61392.1
DDS 22E-1_chr1_2	9577	9759	HicA	TA system compound	ADF59182.1
VC7848_chr1_1	5764	7080	MFS transporter	drug resistance/virulence	WP_011548265.1
VC12802_chr2_2	1499	2896	MFS transporter	drug resistance/virulence	WP_077217595.1

* In region HI2424_chr1_2 two different MFS transporters were found.

## Supplementary Materials

The following are available online at http://www.mdpi.com/1999-4915/10/6/297/s1: Table S1: Prophages BBC, Table S2: Method comparison 895_chr1_1, Table S3: Method comparison 895_chr1_2, Table S4: Method comparison 895_chr1_3, Table S5: Method comparison 895_chr1_7, Table S6: Method comparison 895_chr1_9, Table S7: Method comparison 895_chr2_1, Table S8: Method comparison CR 318_chr1_1, Table S9: Method comparison DDS 22E-1_chr1_2, Table S9: Method comparison DWS 37E-2_chr1_1, Table S11: Method comparison FL-5-3-30-S1-D7_chr1_1, Table S12: Method comparison J2315_chr1_1, Table S13: Method comparison J2315_chr2_1, Table S14: Method comparison MC0-3_chr1_1, Table S15: Method comparison MSMB384WGS_chr1_2, Table S16: Method comparison VC7848_chr1_2, Table S17: Method comparison VC12308_chr1_1, Table S18: Prophage presence complete, Table S19: CRISPR regions, Supplementary data 1: RC 895_chr1_1, Supplementary data 2: RC 895_chr1_2, Supplementary data 3: RC 895_chr1_3, Supplementary data 4: RC 895_chr1_7, Supplementary data 5: RC 895_chr1_9, Supplementary data 6: RC 895_chr2_1, Supplementary data 7: RC CR 318_chr1_1, Supplementary data 8: RC DDS 22E-1_chr1_2, Supplementary data 9: RC DWS 37E-2_chr1_1, Supplementary data 10: RC FL-5-3-30-S1-D7_chr1_2, Supplementary data 11: RC H111_chr1_3, Supplementary data 12: RC J2315_chr1_1, Supplementary data 13: RC J2315_chr2_1, Supplementary data 14: RC MC0-3_chr1_1, Supplementary data 15: RC MSMB384WGS_chr1_2, Supplementary data 16: RC VC7848_chr1_2, Supplementary data 17: RC VC12308_chr1_1, Figure S1: J2315_chr2_1 linear maps, Figure S2: H111_chr1_3 linear maps, Figure S3: DWS 37E-2_chr1_1 linear maps, Figure S4: VC12308_chr1_1 linear maps, Figure S5: FL-5-3-30-S1-D7_chr1_2 linear maps, Figure S6: 895_chr1_1 linear maps, Figure S7: 895_chr1_2 linear maps, Figure S8: 895_chr1_3 linear maps, Figure S9: 895_chr1_7 linear maps, Figure S10: 895_chr1_9 linear maps, Figure S11: 895_chr2_1 linear maps, Figure S12: MSMB384WGS_chr1_2 linear maps, Figure S13: CR 318_chr1_1 linear maps, Figure S14: MC0-3_chr1_1 linear maps, Figure S15: DDS 22E-1chr1_2 linear maps, Figure S16: VC7848_chr1_2 linear maps.
