# Supplementary material for: Burkholderia cenocepacia Prophages—Prevalence, Chromosome Location and Major Genes Involved
Source: Viruses. 2018 May 31;10(6):297. doi: 10.3390/v10060297 (PMC6024312; doi:10.3390/v10060297)
Supplement: Supplementary file 1 [file viruses-10-00297-s001.zip › viruses-297954-r2-supplementary OK/Supplementary data/Region Characteristics Cards/Supplementary_data_1_RC_895_chr1_1.docx]

| **Region characteristics** | | | |
| --- | --- | --- | --- |
| Phage name: | 895_chr1_1 | | |
| Size (nt): | 37,913 | | |
| Type: | Prophage | | |
| Taxonomical affiliation (homology based): | Order: *Caudovirales*  Family: *Myoviridae*  Genus: *Bcepmuvirus* | | |
| Number of annotated open reading frames (ORF): | 50 | | |
| Number of annotated regulatory sequences: | Terminators: | 1 | |
|  | Promoters: | 0 | |
|  | tRNA: | 0 | |
| Derivation: | Host: | | *Burkholderia cenocepacia* 895  chromosome 1 |
|  | Sequence origin (database) | | NCBI |
|  | Accession number/version: | | NZ_CP015036.1 |
|  | Localization in genome: | | 2752594…2790507 |
|  | Additional information: | | - |
| Additional information: | - bacteriophage homologous to temperate phages *Burkholderia* phage phiE255 (NC_009237.1) and *Burkholderia* phage BcepMu (NC_005882.1)  - potential lytic cassette was found in position 11190..12397  - downstream of phage sequence lies protein coding antibiotic resistance. Even though that (theoretically) it doesn’t belong to prophage genome, its localization may suggest:  a) possibility of transferring antibiotic resistance by phage (HGT)  b) artifactual derivation of resistance factors (homologous phages are building in similar/same locations in host genome) | | |

| **Annotation** | | | | | |
| --- | --- | --- | --- | --- | --- |
| **#** | **Strand** | **Start** | **End** | **Length (nt)** | **Product** |
| 1 | - | 1 | 372 | 372 | DNA-binding protein RdgB |
| 2 | - | 369 | 785 | 417 | hypothetical protein |
| 3 | - | 1113 | 1385 | 273 | hypothetical protein |
| 4 | - | 1449 | 2066 | 618 | hypothetical protein |
| 5 | - | 2190 | 2612 | 423 | hypothetical protein |
| 6 | - | 2648 | 2977 | 330 | hypothetical protein |
| 7 | - | 2979 | 4193 | 1215 | hypothetical protein |
| 8 | - | 4190 | 5989 | 1800 | integrase |
| 9 | - | 6007 | 6948 | 942 | hypothetical protein |
| 10 | - | 6960 | 7268 | 309 | transcriptional regulator |
| 11 | - | 7265 | 7744 | 480 | hypothetical protein |
| 12 | + | 7865 | 8101 | 237 | hypothetical protein |
| 13 | - | 8150 | 8392 | 243 | hypothetical protein |
| 14 | + | 8520 | 8936 | 417 | cro/cI repressor transcription regulator |
| 15 | + | 8933 | 9706 | 774 | hypothetical protein |
| 16 | + | 9949 | 10485 | 537 | hypothetical protein |
| 17 | + | 10525 | 10773 | 249 | hypothetical protein |
| 18 | + | 10840 | 11187 | 348 | hypothetical protein |
| 19 | + | 11190 | 11801 | 612 | putative soluble lytic murein transglycosylase |
| 20 | + | 11798 | 12397 | 600 | Rz |
| 21 | + | 12394 | 12732 | 339 | hypothetical protein |
| 22 | + | 12729 | 13061 | 333 | hypothetical protein |
| 23 | + | 13063 | 13608 | 546 | hypothetical protein |
| 24 | + | 13605 | 15107 | 1503 | portal protein |
| 25 | + | 15104 | 16579 | 1476 | hypothetical protein |
| 26 | + | 16572 | 17408 | 837 | virion morphogenesis protein (F) |
| 27 | + | 17405 | 17932 | 528 | Phage tail protein |
| 28 | + | 18135 | 19265 | 1131 | Phage capsid scaffolding protein (GPO) serine peptidase |
| 29 | + | 19308 | 20234 | 927 | Phage major capsid protein E |
| 30 | + | 20309 | 20641 | 333 | conserved hypothetical protein |
| 31 | + | 20643 | 21095 | 453 | hypothetical protein |
| 32 | + | 21095 | 21559 | 465 | hypothetical protein |
| 33 | + | 21556 | 21801 | 246 | hypothetical protein |
| 34 | + | 21805 | 23238 | 1434 | Phage tail sheath protein |
| 35 | + | 23241 | 23765 | 525 | Phage tail tube protein FII |
| 36 | + | 23890 | 24219 | 426 | Phage tail assembly chaperone proteins |
| 37 | + | 24146 | 24349 | 129 | putative lambda G-pre-tape measure frameshift protein |
| 38 | - | 24352 | 24594 | 243 | hypothetical protein |
| 39 | + | 24624 | 27230 | 2607 | Minor tail protein |
| 40 | + | 27239 | 28129 | 891 | GpU protein |
| 41 | + | 28129 | 28338 | 210 | Tail protein X |
| 42 | + | 28326 | 29531 | 1206 | Tail protein GpD |
| 43 | + | 29528 | 30130 | 603 | Baseplate protein |
| 44 | + | 30184 | 30537 | 354 | Baseplate assembly protein GpW |
| 45 | + | 30534 | 31685 | 1152 | Baseplate J-like protein |
| 46 | + | 31678 | 32259 | 582 | Tail fiber protein |
| 47 | + | 32259 | 34706 | 2448 | Tail fiber protein |

| **Terminators** | | | |
| --- | --- | --- | --- |
| **Strand** | **Start** | **End** | **Sequence** |
| + | 23796 | 23812 | GGCCCGCTTCGGCGGGC |
