# Supplementary material for: Burkholderia cenocepacia Prophages—Prevalence, Chromosome Location and Major Genes Involved
Source: Viruses. 2018 May 31;10(6):297. doi: 10.3390/v10060297 (PMC6024312; doi:10.3390/v10060297)
Supplement: Supplementary file 1 [file viruses-10-00297-s001.zip › viruses-297954-r2-supplementary OK/Supplementary data/Region Characteristics Cards/Supplementary_data_10_RC_FL-5-3-30-S1-D7_chr1_2.docx]

| **Region characteristics** | | | |
| --- | --- | --- | --- |
| Phage name: | FL-5-3-30-S1-D7_chr1_2 | | |
| Size (nt): | 44063 | | |
| Type: | Prophage | | |
| Taxonomical affiliation (homology based): | Order: *Caudovirales*  Family: *Siphoviridae* | | |
| Number of annotated open reading frames (ORF): | 51 | | |
| Number of annotated regulatory sequences: | Terminators: | 11 | |
|  | Promoters: | 0 | |
|  | tRNA: | 1 | |
| Derivation: | Host: | | *Burkholderia cenocepacia* FL-5-3-30-S1-D7,  chromosome 1 |
|  | Sequence origin (database) | | NCBI |
|  | Accession number/version: | | CP013397.1 |
|  | Localization in genome: | | 2017976..2062038 |
|  | Additional information: | | - |
| Additional information: | - Phage poses sequences which may serve as *cos* sites  - integrase identified  - terminase identified  - complete lytic cassette was found in position  - *virulence protein E* (WP_040144422.1), is located inside the phage genome, not in the neighborhood, as it was in case of DWS 37E-2_chr1_1 (position 32794..35301)  - trifling homology (2-4%) suggest derivation to *Siphoviridae*  - of the genes that were found in region:  a) genes show homology with known phage genes  b) genes are distinctive for phages, although with no homology to viral sequences in the database (green)  c) 31 genes with homology to bacterial genes (blue) | | |

| **Annotation** | | | | | |
| --- | --- | --- | --- | --- | --- |
| **#** | **Strand** | **Start** | **End** | **Length (nt)** | **Product** |
| 1 | - | 1 | 960 | 960 | alpha/beta hydrolase |
| 2 | - | 1128 | 1607 | 480 | thioesterase |
| 3 | - | 1604 | 1900 | 297 | stress responsive protein |
| - | x | 2016 | 2042 | 24 | attR, right cohesive end |
| 4 | + | 2097 | 3311 | 1215 | integrase |
| 5 | - | 3894 | 4094 | 201 | hypothetical protein |
| 6 | - | 4104 | 4820 | 717 | hypothetical protein |
| 7 | - | 4833 | 5309 | 477 | hypothetical protein |
| 8 | - | 5332 | 5802 | 471 | hypothetical protein |
| 9 | - | 5792 | 6289 | 498 | hypothetical protein |
| 10 | - | 6296 | 6547 | 252 | hypothetical protein |
| 11 | - | 6901 | 7689 | 789 | DNA adenine methylase |
| 12 | - | 7860 | 8354 | 495 | Rz protein |
| 13 | - | 8351 | 8845 | 495 | endolysin |
| 14 | - | 8848 | 9132 | 285 | holin |
| 15 | - | 9208 | 10257 | 1050 | tail protein |
| 16 | - | 10448 | 11326 | 879 | oxidoreductase |
| 17 | - | 11337 | 13787 | 2451 | phage tail protein |
| 18 | - | 13868 | 14170 | 303 | hypothetical protein |
| 19 | - | 14278 | 14781 | 504 | phage tail protein |
| 20 | - | 14792 | 15961 | 1170 | tail sheath protein |
| 21 | - | 16046 | 16798 | 753 | tail fiber assembly protein |
| 22 | - | 16814 | 18781 | 1968 | tail fiber protein |
| 23 | - | 18769 | 19347 | 579 | tail protein |
| 24 | - | 19337 | 20233 | 897 | baseplate J-like protein |
| 25 | - | 20230 | 20565 | 336 | baseplate assembly protein |
| 26 | - | 20565 | 20765 | 201 | hypothetical protein |
| 27 | + | 20879 | 21670 | 792 | hypothetical protein |
| 28 | - | 21673 | 22356 | 684 | baseplate assembly protein |
| 29 | - | 22360 | 22878 | 519 | hypothetical protein |
| 30 | - | 22868 | 23398 | 531 | phage tail protein |
| 31 | - | 23401 | 23688 | 288 | hypothetical protein |
| 32 | - | 23690 | 24685 | 996 | major capsid protein E |
| 33 | - | 24759 | 25103 | 345 | head decoration protein |
| 34 | - | 25134 | 26216 | 1083 | peptidase S14 |
| 35 | - | 26213 | 27706 | 1494 | portal protein |
| 36 | - | 27703 | 27909 | 207 | hypothetical protein |
| 37 | - | 27923 | 29842 | 1920 | terminase large subunit |
| 38 | - | 29871 | 30440 | 570 | hypothetical protein |
| 39 | + | 30857 | 31225 | 369 | hypothetical protein |
| 40 | - | 31358 | 31552 | 195 | hypothetical protein |
| 41 | - | 31800 | 32573 | 774 | hypothetical protein |
| 42 | - | 32794 | 35301 | 2508 | virulence protein E |
| 43 | - | 35562 | 35945 | 384 | hypothetical protein |
| 44 | + | 35944 | 36486 | 543 | hypothetical protein |
| 45 | + | 36820 | 37566 | 747 | helix-turn-helix domain-containing protein |
| 46 | + | 37737 | 38015 | 279 | hypothetical protein |
| 47 | + | 38053 | 38427 | 375 | beta-hexosaminidase |
| 48 | + | 38427 | 40031 | 1605 | pyridoxal phosphate biosynthetic protein PdxJ |
| 49 | + | 40133 | 40474 | 342 | hypothetical protein |
| 50 | - | 41644 | 42729 | 1086 | hypothetical protein |
| 51 | - | 42863 | 43285 | 423 | pilus assembly protein TadE |
| - | x | 44037 | 44063 | 24 | attL, left cohesive end |

| **Terminators** | | | |
| --- | --- | --- | --- |
| **Strand** | **Start** | **End** | **Sequence** |
| + | 2051 | 2087 | GGGGGTATCATTTCGGCCATCCAGAGACGATACCCCC |
| - | 2051 | 2087 | GGGGGTATCGTCTCTGGATGGCCGAAATGATACCCCC |
| - | 9160 | 9186 | GGCCGCTCACGGGCAACCGGAGCGGCC |
| + | 9160 | 9186 | GGCCGCTCCGGTTGCCCGTGAGCGGCC |
| - | 14192 | 14212 | TGGCGAGCCGACGGCTCGCCA |
| - | 15985 | 16002 | GCCGCTCCGATGAGCGGC |
| - | 16014 | 16039 | CGACGCCACTCGTCCGGGTGGCGTCG |
| - | 24718 | 24738 | GGCCACGCCGACCGCGTGGCC |
| - | 30481 | 30500 | TGCGGGGGCTCGCACCCGCG |
| - | 31574 | 31599 | GCCCTGAGTGCGAAAGCCCTCAGGGC |
| + | 40819 | 40835 | TCCCGCTTCGGCGGGA |
