# Supplementary material for: Burkholderia cenocepacia Prophages—Prevalence, Chromosome Location and Major Genes Involved
Source: Viruses. 2018 May 31;10(6):297. doi: 10.3390/v10060297 (PMC6024312; doi:10.3390/v10060297)
Supplement: Supplementary file 1 [file viruses-10-00297-s001.zip › viruses-297954-r2-supplementary OK/Supplementary data/Region Characteristics Cards/Supplementary_data_11_RC_H111_chr1_3.docx]

| **Region characteristics** | | | |
| --- | --- | --- | --- |
| Phage name: | H111_chr1_3 | | |
| Size (nt): | 38390 | | |
| Type: | Artifact region | | |
| Taxonomical affiliation (homology based): | - | | |
| Number of annotated open reading frames (ORF): | 49 | | |
| Number of annotated regulatory sequences: | Terminators: | - | |
|  | Promoters: | - | |
|  | tRNA: | - | |
| Derivation: | Host: | | *Burkholderia cenocepacia* H111,  chromosome 1 |
|  | Sequence origin (database) | | NCBI |
|  | Accession number/version: | | NZ_HG938370.1 |
|  | Localization in genome: | | 1609303..1647690 |
|  | Additional information: | | Even though Phaster recognize region as complete phage, annotation shows that it is probably non-functional virus. Genes found in this region, often show homology to phages from various taxonomical groups and specific to hosts other than *Burkholderia.* |
| Additional information: | - Region contains sequences which might have served as *cos* sites  - Phaster included bacterial tRNA to the region, in which in closest neighborhood lies *attR*  *­*- homologue of lysozyme was found (30307..30804)  - of the genes that were found in region:  a) 18 genes show homology with known phage genes  b) 7 genes are distinctive for phages, although with no homology to viral sequences in the database (green)  c) 24 genes with homology to bacterial genes (not mentioned in annotation table) | | |

| **Annotation** | | | | | | | | | | | |
| --- | --- | --- | --- | --- | --- | --- | --- | --- | --- | --- | --- |
| **#** | **Strand** | **Start** | **End** | **Length (nt)** | **Product** | **Homology** | | | | |  |
|  |  |  |  |  |  | Phage name | A/N | QC % | Ident% |  |  |
| 1 | - | 1261 | 1560 | 279 | DNA binding protein | *Burkholderia* phage KS5 | YP_004306375.1 | 84 | 54 |  |  |
| 2 | + | 2002 | 2319 | 318 | hypothetical protein | *Burkholderia* virus BcepF1 | YP_001039691.1 | 87 | 64 |  |  |
| 3 | + | 2350 | 3213 | 864 | putative chromosome partitioning protein | *Burkholderia* virus phiE125 | NP_536415.1 | 82 | 61 |  |  |
| 4 | + | 4746 | 5291 | 546 | hypothetical protein | *Burkholderia* virus phi6442 | YP_001111140.1 | 100 | 45 |  |  |
| 5 | + | 5744 | 6085 | 342 | hypothetical protein | *Burkholderia* phage Bcep176 | YP_355340.1 | 100 | 88 |  |  |
| 6 | + | 6088 | 6420 | 333 | hypothetical protein | *Burkholderia* virus phi1026b | NP_945106.1 | 98 | 70 |  |  |
| 7 | + | 6417 | 6656 | 240 | hypothetical protein | *Burkholderia* virus phi6442 | YP_001111144.1 | 98 | 45 |  |  |
| 8 | + | 6656 | 6913 | 258 | hypothetical protein | *Burkholderia* virus Bcep22 | NP_944247.1 | 90 | 47 |  |  |
| 9 | + | 7823 | 8515 | 432 | minor tail protein | *Rhodobacter* phage RcRhea | YP_009213512.1 | 95 | 32 |  |  |
| 10 | + | 9230 | 11269 | 2040 | terminase large subunit | uncultured Mediterranean phage uvMED | BAR26880.1 | 85 | 47 |  |  |
| x | + | 11311 | 11547 | 237 | phage head-tail adapter protein | *Burkholderia cenocepacia* | KWF74592.1 | 100 | 100 |  |  |
| 11 | + | 11544 | 13199 | 1656 | portal protein | *Xylella* phage Sano | AHB12085.1 | 87 | 36 |  |  |
| x | + | 13221 | 14117 | 897 | serine peptidase | n/d | NP_006495264.1 | 100 | 100 |  |  |
| x | + | 14754 | 15167 | 588 | head decoration protein | *Burkholderia cenocepacia* | WP_043204876.1 | 100 | 100 |  |  |
| 12 | + | 15240 | 16286 | 1029 | major capsid protein | *Xylella* phage Sano | AHB12081.1 | 98 | 33 |  |  |
| 13 | + | 17663 | 19153 | 1491 | tail sheath protein | *Enterobacteria* phage SfI | YP_009147459.1 | 99 | 44 |  |  |
| x | + | 19223 | 19597 | 375 | phage tail protein | *Burkholderia cenocepacia* | WP_006495255.1 | 100 | 100 |  |  |
| x | + | 20325 | 21749 | 1425 | multidrug DMT transporter | *Burkholderia cenocepacia* | WP_006495253.1 | 100 | 100 |  |  |
| 14 | + | 23415 | 24557 | 1143 | tail protein | *Enterobacteria* phage SfI | YP_009147464.1 | 90 | 29 |  |  |
| 15 | + | 25124 | 25570 | 1447 | tail protein | *Escherichia* virus Mu | NP_050650.1 | 97 | 43 |  |  |
| 16 | + | 25572 | 26735 | 447 | base plate J protein | *Salmonella* phage 118970_sal3 | YP_009324807.1 | 99 | 32 |  |  |
| 17 | + | 26742 | 27338 | 597 | tail protein | *Enterobacteria* phage phiP27 | NP_543105.1 | 94 | 33 |  |  |
| x | + | 27347 | 28888 | 1542 | tail fiber protein | *Burkholderia cenocepacia* | CDN60052.1 | 100 | 100 |  |  |
| x | + | 30307 | 30804 | 418 | glycosyl hydrolase | *Burkholderia sp.* | WP_029227429.1 | 100 | 100 |  |  |
| 18 | + | 31343 | 31822 | 480 | Rz | *Burkholderia* virus BcepC6B | YP_024943.1 | 97 | 80 |  |  |
| 19 | - | 32955 | 34034 | 1080 | integrase | *Burkholderia* phage AH2 | YP_006561121.1 | 98 | 55 |  |  |
