# Supplementary material for: Burkholderia cenocepacia Prophages—Prevalence, Chromosome Location and Major Genes Involved
Source: Viruses. 2018 May 31;10(6):297. doi: 10.3390/v10060297 (PMC6024312; doi:10.3390/v10060297)
Supplement: Supplementary file 1 [file viruses-10-00297-s001.zip › viruses-297954-r2-supplementary OK/Supplementary data/Region Characteristics Cards/Supplementary_data_12_RC_J2315_chr1_1.docx]

| **Region characteristics** | | | |
| --- | --- | --- | --- |
| Phage name: | J2315_chr1_1 | | |
| Size (nt): | 24997 | | |
| Type: | Prophage | | |
| Taxonomical affiliation (homology based): | Order: *Caudovirales*  Family: *Myoviridae* | | |
| Number of annotated open reading frames (ORF): | 29 | | |
| Number of annotated regulatory sequences: | Terminators: | 0 | |
|  | Promoters: | 0 | |
|  | tRNA: | 0 | |
| Derivation: | Host: | | *Burkholderia cenocepacia* J2315,  chromosome 1 |
|  | Sequence origin (database) | | NCBI |
|  | Accession number/version: | | NC_011000.1 |
|  | Localization in genome: | | 100299..125296 |
|  | Additional information: | | - |
| Additional information: | - Start position of the phage genome has been altered in comparison to Phaster (99160) and moved to the *cos* site  - End position of the phage genome has been altered in comparison to Phaster (130529) and moved to the *cos* site  - sequences which may serve as *cos* sites were found  - potential lysis cassette was found in position #22-25  - of the genes that were found in region:  a) 28 genes show homology with known phage genes  b) 1 genes with homology to bacterial genes (blue) | | |

| **Annotation** | | | | | |
| --- | --- | --- | --- | --- | --- |
| **#** | **Strand** | **Start** | **End** | **Length (nt)** | **Product** |
| x | x | 1 | 31 | 31 | attL |
| 1 | - | 100 | 1176 | 1077 | integrase |
| 2 | + | 1179 | 1451 | 273 | hypothetical protein |
| x | - | 1871 | 4663 | 2793 | hypothetical protein |
| 3 | - | 4669 | 4926 | 258 | hypothetical protein |
| 4 | - | 5054 | 5302 | 249 | ogr/Delta-like zinc finger family protein |
| 5 | - | 5484 | 5657 | 174 | hypothetical protein |
| 6 | + | 5790 | 6281 | 492 | repressor |
| 7 | + | 6853 | 8385 | 1533 | reverse transcriptase |
| 8 | - | 8997 | 10067 | 1071 | bacteriophage late control gene D protein |
| 9 | - | 10064 | 10495 | 432 | fels-2 protein |
| 10 | - | 10518 | 13088 | 2571 | tail tape measure protein (T) |
| 11 | - | 13104 | 13217 | 114 | tail protein |
| 12 | - | 13226 | 13597 | 372 | tail protein E |
| 13 | - | 13674 | 14183 | 510 | major tail tube protein |
| 14 | - | 14212 | 15384 | 1173 | tail sheath protein |
| 15 | - | 15439 | 16173 | 735 | tail fiber assembly protein |
| 16 | - | 16189 | 18840 | 2652 | tail fiber protein |
| 17 | - | 18847 | 19389 | 543 | phage tail protein I |
| 18 | - | 19382 | 20296 | 915 | baseplate assembly protein J |
| 19 | - | 20293 | 20655 | 363 | baseplate assembly protein W |
| 20 | - | 20652 | 21338 | 687 | baseplate assembly V family protein |
| 21 | - | 21847 | 22296 | 450 | tail completion protein R |
| 22 | - | 22413 | 22853 | 441 | Rz |
| 23 | - | 22850 | 23707 | 858 | endolysin |
| 24 | - | 23704 | 23970 | 267 | holin |
| 25 | - | 23972 | 24316 | 345 | holin |
| 26 | - | 24333 | 24539 | 207 | tail protein X |
| x | x | 24937 | 24967 | 31 | attR |
