# Supplementary material for: Burkholderia cenocepacia Prophages—Prevalence, Chromosome Location and Major Genes Involved
Source: Viruses. 2018 May 31;10(6):297. doi: 10.3390/v10060297 (PMC6024312; doi:10.3390/v10060297)
Supplement: Supplementary file 1 [file viruses-10-00297-s001.zip › viruses-297954-r2-supplementary OK/Supplementary data/Region Characteristics Cards/Supplementary_data_13_RC_J2315_chr2_1.docx]

| **Region characteristics** | | | |
| --- | --- | --- | --- |
| Phage name: | J2315_chr2_1 | | |
| Size (nt): | 46822 | | |
| Type: | Artifact region | | |
| Taxonomical affiliation (homology based): | - | | |
| Number of annotated open reading frames (ORF): | 76 | | |
| Number of annotated regulatory sequences: | Terminators: | - | |
|  | Promoters: | - | |
|  | tRNA: | - | |
| Derivation: | Host: | | *Burkholderia cenocepacia* J2315,  chromosome 2 |
|  | Sequence origin (database) | | NCBI |
|  | Accession number/version: | | NC_011001.1 |
|  | Localization in genome: | | 1140168..1186990 |
|  | Additional information: | | Even though Phaster recognize region as complete phage, annotation shows that it is probably non-functional virus. Genes found in this region, often show homology to phages from various taxonomical groups and specific to hosts other than *Burkholderia.* |
| Additional information: | - Region contains sequences which might have served as *cos* sites  - Phaster included bacterial tRNA to the region, in which in closest neighborhood lies *attL* (excluded in manual annotation)  *­­*- manual annotation have shown low homology to virus proteins database  - of the genes that were found in region:  a) 35 genes show homology with known phage genes  b) 9 genes are distinctive for phages, although with no homology to viral sequences in the database (green)  c) 33 genes with homology to bacterial genes (not mentioned in annotation table) | | |

| **Annotation** | | | | | | | | | | | |
| --- | --- | --- | --- | --- | --- | --- | --- | --- | --- | --- | --- |
| **#** | **Strand** | **Start** | **End** | **Length (nt)** | **Product** | **Homology** | | | | |  |
|  |  |  |  |  |  | Phage name | A/N | QC % | Ident% |  |  |
| 1 | + | 1 | 15 | 15 | attL | x | x | x | x |  |  |
| X | - | 103 | 1092 | 990 | integrase | *-* | WP_012493219.1 | 100 | 100 |  |  |
| 2 | - | 1637 | 2158 | 522 | hypothetical protein | *Pseudomonas phage vB_PaeM_G1* | ARW57287.1 | 100 | 99 |  |  |
| X | - | 3542 | 3784 | 243 | XRE family transcriptional regulator | *-* | - | 100 | 100 |  |  |
| 3 | - | 5997 | 6602 | 606 | hypothetical protein | *Escherichia phage vB_EcoM_ECO1230-10* | YP_009168925.1 | 87 | 48 |  |  |
| 4 | - | 6728 | 7408 | 681 | endonuclease | *Paracoccus phage Shpa* | AKG94550.1 | 97 | 38 |  |  |
| X | - | 9616 | 9783 | 168 | phage membrane protein | *-* | CAR54896.1 | 100 | 100 |  |  |
| X | - | 12317 | 12700 | 384 | type II toxin-antitoxin system | *-* | WP_006488862.1 | 100 | 100 |  |  |
| X | - | 12971 | 14503 | 1533 | group II intron reverse transcriptase/maturase | *-* | WP_012493229.1 | 100 | 100 |  |  |
| 5 | + | 15671 | 15925 | 255 | antirepressor | *Enterobacteria phage mEp043 c-1* | YP_007111551.1 | 86 | 55 |  |  |
| 6 | + | 16524 | 16799 | 276 | similar DNA binding protein | *Burkholderia phage vB_BceM_AP3* | AKA61162.1 | 91 | 57 |  |  |
| 7 | + | 16784 | 17713 | 930 | DUF1376 domain-containing protein | *-* | WP_006488817.1 | 100 | 99 |  |  |
| 8 | + | 18299 | 18769 | 471 | endodeoxyribonuclease RusA | *Burkholderia phage Bups phi1* | ABY40522.1 | 100 | 80 |  |  |
| 9 | + | 18780 | 19109 | 330 | hypothetical protein | *Burkholderia phage Bups phi1* | ABY40523.1 | 85 | 62 |  |  |
| 10 | + | 19106 | 19699 | 594 | hypothetical protein | *Bups phi1* | ABY40524.1 | 96 | 78 |  |  |
| 11 | + | 20345 | 21880 | 1536 | TerL | *Klebsiella phage JD001* | YP_007392855.1 | 96 | 46 |  |  |
| 12 | + | 21877 | 23460 | 1584 | head morphogenesis protein | *Burkholderia phage Bups phi1* | ABY40531.1 | 99 | 77 |  |  |
| 13 | + | 23390 | 24040 | 651 | head morphogenesis protein | *-* | ONR50492.1 | 100 | 100 |  |  |
| 14 | + | 24042 | 25358 | 1317 | hypothetical protein | *Acinetobacter phage Ab105-1phi* | ALJ99064.1 | 99 | 41 |  |  |
| 15 | + | 25371 | 25859 | 489 | hypothetical protein | *Acinetobacter phage Ab105-1phi* | ALJ99065.1 | 100 | 56 |  |  |
| 16 | + | 25870 | 26907 | 1038 | hypothetical protein | *Acinetobacter phage Ab105-1phi* | ALJ99066.1 | 98 | 68 |  |  |
| 17 | + | 26917 | 27342 | 426 | hypothetical protein | *Burkholderia phage Bups phi1* | ABY40557.1 | 84 | 41 |  |  |
| 18 | + | 27398 | 27781 | 384 | hypothetical protein | *Burkholderia phage Bups phi1* | ABY40558.1 | 100 | 75 |  |  |
| 19 | + | 27810 | 28292 | 483 | hypothetical protein | *Burkholderia phage Bups phi1* | ABY40559.1 | 99 | 86 |  |  |
| 20 | + | 28296 | 28667 | 372 | hypothetical protein | *Burkholderia phage Bups phi1* | ABY40561.1 | 100 | 89 |  |  |
| 21 | + | 28672 | 29262 | 591 | hypothetical protein | *Burkholderia phage Bups phi1* | ABY40562.1 | 99 | 87 |  |  |
| 22 | + | 29272 | 31083 | 1812 | hypothetical protein | *Acinetobacter phage Ab105-1phi* | ALJ99072.1 | 77 | 44 |  |  |
| 23 | + | 31101 | 31541 | 441 | hypothetical protein | *Acinetobacter phage Ab105-1phi* | ALJ99073.1 | 100 | 58 |  |  |
| 24 | + | 31544 | 31996 | 453 | hypothetical protein | *Acinetobacter phage Ab105-1phi* | ALJ99074.1 | 91 | 37 |  |  |
| X | + | 32171 | 33958 | 1788 | glucosaminidase | *-* | WP_006488896.1 | 100 | 100 |  |  |
| 25 | + | 33955 | 34560 | 606 | hypothetical protein | *Salmonella phage SEN34* | YP_009191623.1 | 85 | 37 |  |  |
| 26 | + | 34557 | 34874 | 318 | hypothetical protein | *Salmonella phage SEN34* | YP_009191624.1 | 93 | 47 |  |  |
| 27 | + | 35507 | 36517 | 1011 | hypothetical protein | *Burkholderia phage Bups phi1* | ABY40537.1 | 86 | 60 |  |  |
| 28 | - | 36514 | 36942 | 429 | hypothetical protein | *Acinetobacter phage Ab105-1phi* | ALJ99061.1 | 87 | 59 |  |  |
| 29 | + | 36941 | 37732 | 792 | hypothetical protein | *Burkholderia phage Bups phi1* | ABY40539.1 | 100 | 54 |  |  |
| 30 | + | 37740 | 38090 | 351 | hypothetical protein | *Burkholderia phage Bups phi1* | ABY40544.1 | 100 | 71 |  |  |
| 31 | + | 38087 | 39274 | 1188 | hypothetical protein | *Burkholderia phage Bups phi1* | ABY40545.1 | 98 | 69 |  |  |
| 32 | + | 39276 | 39992 | 717 | hypothetical protein | *Burkholderia phage Bups phi1* | ABY40546.1 | 90 | 62 |  |  |
| 33 | + | 40049 | 41047 | 999 | tail protein | *Burkholderia phage Bups phi1* | ABY40547.1 | 61 | 44 |  |  |
| X | + | 41659 | 43215 | 1557 | pectate lyase | *-* | WP_006488897.1 | 100 | 100 |  |  |
| X | + | 43673 | 44059 | 387 | phage transmembrane protein | *-* | CAR54949.1 | 100 | 100 |  |  |
| 34 | + | 44056 | 44628 | 573 | glycoside hydrolase family 19 protein | *Pseudomonas phage phiPSA1* | YP_009043563.1 | 98 | 41 |  |  |
| X | + | 46017 | 46613 | 597 | lipase | *-* | WP_006488875.1 | 100 | 100 |  |  |
| 35 | + | 46809 | 46823 | 15 | attR | x | x | x | x |  |  |
