# Supplementary material for: Burkholderia cenocepacia Prophages—Prevalence, Chromosome Location and Major Genes Involved
Source: Viruses. 2018 May 31;10(6):297. doi: 10.3390/v10060297 (PMC6024312; doi:10.3390/v10060297)
Supplement: Supplementary file 1 [file viruses-10-00297-s001.zip › viruses-297954-r2-supplementary OK/Supplementary data/Region Characteristics Cards/Supplementary_data_14_RC_MC0-3_chr1_1.docx]

| **Region characteristics** | | | |
| --- | --- | --- | --- |
| Phage name: | MC0-3_chr1_1 | | |
| Size (nt): | 40,720 | | |
| Type: | Prophage | | |
| Taxonomical affiliation (homology based): | Order: *Caudovirales*  Family: *Myoviridae*  Genus: *Peduovirinae* | | |
| Number of annotated open reading frames (ORF): | 44 | | |
| Number of annotated regulatory sequences: | Terminators: | 0 | |
|  | Promoters: | 0 | |
|  | tRNA: | 0 | |
| Derivation: | Host: | | *Burkholderia cenocepacia* MC0-3  chromosome 1 |
|  | Sequence origin (database) | | NCBI |
|  | Accession number/version: | | NC_010508.1 |
|  | Localization in genome: | | 198439…237310 |
|  | Additional information: | | - |
| Additional information: | - potential cos-sites found  - prophage integrates in tRNA-Arg  - bacteriophage homologous to temperate phage *Burkholderia* phage vB_BceM_AP3 (KP966108.1)  - potential lytic cassette was found in position #28-#31 (24624...26599) | | |

| **Annotation** | | | | | |
| --- | --- | --- | --- | --- | --- |
| **#** | **Strand** | **Start** | **End** | **Length (nt)** | **Product** |
| x | x | 1 | 45 | 45 | attL |
| 1 | - | 121 | 1230 | 1110 | integrase |
| 2 | - | 1557 | 2123 | 567 | hypothetical protein |
| 3 | - | 2120 | 4912 | 2793 | hypothetical protein |
| 4 | - | 4915 | 5169 | 255 | hypothetical protein |
| 5 | - | 5166 | 5528 | 363 | hypothetical protein |
| 6 | - | 5531 | 5686 | 156 | hypothetical protein |
| 7 | - | 5691 | 5885 | 195 | hypothetical protein |
| 8 | - | 5929 | 6123 | 195 | hypothetical protein |
| 9 | - | 6128 | 6322 | 195 | hypothetical protein |
| 10 | - | 6411 | 6659 | 249 | hypothetical protein |
| 11 | - | 6687 | 6875 | 189 | hypothetical protein |
| x | - | 7112 | 7297 | 186 | hypothetical protein |
| x | + | 7905 | 7914 | 10 | transcriptional regulator |
| x | - | 7905 | 8285 | 381 | LysR family transcriptional regulator |
| 12 | - | 8752 | 9903 | 1152 | late control geneD protein |
| 13 | - | 10342 | 13101 | 2760 | tail tape measure protein (T) |
| 14 | - | 13098 | 13217 | 120 | P2 GpE family protein |
| 15 | - | 13217 | 13534 | 318 | tail protein |
| 16 | - | 13561 | 14070 | 510 | major tail tube protein |
| 17 | - | 14100 | 15272 | 1173 | tail sheath protein |
| 18 | - | 15384 | 16133 | 750 | DNA methylase N-4 |
| 19 | - | 16443 | 16781 | 339 | hypothetical protein |
| 20 | - | 16848 | 17276 | 429 | hypothetical protein |
| 21 | - | 17273 | 18982 | 1710 | tail fiber protein |
| 22 | - | 18988 | 19554 | 567 | tail protein |
| 23 | - | 19532 | 20437 | 906 | baseplate assembly protein |
| 24 | - | 20434 | 20799 | 366 | baseplate assembly protein |
| 25 | - | 20796 | 21500 | 705 | baseplate assembly protein |
| x | - | 21780 | 23501 | 1722 | hypothetical protein |
| 26 | - | 23768 | 24217 | 450 | virion morphogenesis protein |
| 27 | - | 24217 | 24627 | 411 | tail completion protein |
| 28 | - | 24624 | 25115 | 492 | i-spanin |
| 29 | - | 25112 | 25912 | 801 | endolysin |
| 30 | - | 25905 | 26225 | 321 | holin |
| 31 | - | 26225 | 26599 | 375 | antiholin |
| 32 | - | 26602 | 26814 | 213 | tail protein |
| 33 | - | 26814 | 27296 | 483 | head completion protein |
| 34 | - | 27401 | 28087 | 687 | small terminase subunit |
| 35 | - | 28084 | 29103 | 1020 | major capsid protein |
| 36 | - | 29140 | 29958 | 819 | capsid scaffolding protein |
| 37 | + | 30103 | 31854 | 1752 | terminase ATPase subunit (P) |
| 38 | + | 31854 | 32906 | 1053 | portal vertex protein |
| 38 | - | 32934 | 33662 | 729 | hypothetical protein |
| 39 | + | 34028 | 34625 | 598 | hypothetical protein |
| 40 | - | 34625 | 35740 | 1116 | hypothetical protein |
| 41 | - | 35746 | 36516 | 771 | hypothetical protein |
| 42 | - | 36513 | 37049 | 537 | hypothetical protein |
| x | - | 37443 | 38240 | 798 | (p)ppGpp synthetase |
| x | x | 38806 | 38850 | 45 | attR |

| **Terminators** | | | |
| --- | --- | --- | --- |
| **Strand** | **Start** | **End** | **Sequence** |
|  |  |  |  |
