# Supplementary material for: Burkholderia cenocepacia Prophages—Prevalence, Chromosome Location and Major Genes Involved
Source: Viruses. 2018 May 31;10(6):297. doi: 10.3390/v10060297 (PMC6024312; doi:10.3390/v10060297)
Supplement: Supplementary file 1 [file viruses-10-00297-s001.zip › viruses-297954-r2-supplementary OK/Supplementary data/Region Characteristics Cards/Supplementary_data_15_RC_MSMB384WGS_chr1_2.docx]

| **Region characteristics** | | | |
| --- | --- | --- | --- |
| Phage name: | MSMB384WGS_chr1_2 | | |
| Size (nt): | 49010 | | |
| Type: | Artifact region | | |
| Taxonomical affiliation (homology based): | - | | |
| Number of annotated open reading frames (ORF): | 64 | | |
| Number of annotated regulatory sequences: | Terminators: | 10 | |
|  | Promoters: | - | |
|  | tRNA: | - | |
| Derivation: | Host: | | *Burkholderia cenocepacia* MSMB384WGS,  chromosome 1 |
|  | Sequence origin (database) | | NCBI |
|  | Accession number/version: | | NZ_CP013450.1 |
|  | Localization in genome: | | 1394910..1443874 |
|  | Additional information: | | Even though Phaster recognize region as complete phage, annotation shows that it is probably non-functional virus. Genes found in this region, often show homology to phages from various taxonomical groups and specific to hosts other than *Burkholderia.* |
| Additional information: | - of the genes that were found in region:  a) 19 genes show homology with known phage genes  b) 11 genes are distinctive for phages, although with no homology to viral sequences in the database (green)  b) 34 genes with homology to bacterial genes (not mentioned in annotation table)  - directly downstream to the region there are genes taking part in encoding secretion systems  - potential *cos* sites have been located  - the *attL* has a sequence homological to the host tRNA  - often genes possess high disparity in comparison to their homologues (e.g. point mutations, lowering overall score of comparison or introducing changes in stop codons, influencing length of the gene). Having that in mind, it was decided to omit homology of the gene to the viruses database if it was less than 40%. In this circumstances the result from the comparison again bacterial database was taken into consideration if it was more than 40%. | | |

| **Annotation** | | | | | | | | | |
| --- | --- | --- | --- | --- | --- | --- | --- | --- | --- |
| **#** | **Strand** | **Start** | **End** | **Length(nt)** | **Product** | **Homology** | | | |
|  |  |  |  |  |  | Phage name | A/N | QC % | Ident% |
| 1 | 56 | 1150 | - | 1095 | integrase | *Escherichia* phage HK75 | YP_004934132.1 | 93 | 37 |
| 2 | 1359 | 1955 | + | 597 | hypothetical protein | *Pseudomonas* phage F116 | YP_164271.1 | 98 | 38 |
| 3 | 2507 | 2926 | + | 531 | hypothetical protein | *Burkholderia* phage Bcep176 | YP_355385.1 | 100 | 86 |
| 4 | 6043 | 7143 | - | 1101 | putative DNA cytosine methylase | *Burkholderia* virus phiE125] | NP_536413.1 | 99 | 65 |
| 5 | 8184 | 8402 | - | 219 | hypothetical protein | *Burkholderia* phage Bcep176 | YP_355376.1 | 100 | 55 |
| x | 11252 | 11728 | - | 477 | transcriptional regulator | *Burkholderia* *cenocepacia* | WP_059668021.1 | 98 | 60 |
| 7 | 13648 | 13950 | + | 303 | hypothetical protein | *Burkholderia* virus BcepF1 | YP_001039691.1 | 92 | 66 |
| 8 | 14029 | 14853 | + | 825 | putative chromosome partitioning protein | *Burkholderia* virus phi6442 | YP_001111136.1 | 90 | 68 |
| 9 | 14850 | 15863 | + | 1014 | hypothetical protein | *Burkholderia* virus phi1026b | NP_945102.1 | 98 | 57 |
| 10 | 15860 | 16405 | + | 546 | hypothetical protein | *Burkholderia* virus phi6442 | YP_001111140.1 | 100 | 46 |
| x | 16413 | 16805 | + | 393 | winged helix-turn-helix domain-containing protein | *Burkholderia cenocepacia* | WP_060263150.1 | 100 | 100 |
| 11 | 18500 | 18811 | + | 312 | hypothetical protein | *Pseudomonas* phage H66 | AGC34618.1 | 94 | 57 |
| 12 | 19783 | 20472 | + | 690 | minor tail protein | *Rhodobacter* phage RcRhea | YP_009213512.1 | 95 | 43 |
| 13 | 21190 | 23226 | + | 2037 | terminase large subunit | *Synechococcus* phage S-CAM8 | AET72686.1 | 84 | 48 |
| x | 23268 | 23504 | + | 237 | phage head-tail adapter protein | *Burkholderia* *cenocepacia* | KWF74592.1 | 100 | 100 |
| 14 | 23501 | 25156 | + | 1656 | portal protein | *Xylella* phage Sano | AHB12085.1 | 92 | 34 |
| x | 25185 | 26081 | + | 897 | serine peptidase | *Burkholderia cenocepacia* | WP_060263160.1 | 100 | 100 |
| 15 | 27205 | 28251 | + | 1047 | major capsid protein | *Xylella* phage Sano | AHB12081.1 | 98 | 34 |
| 16 | 29628 | 31118 | + | 1491 | tail sheath protein | *Enterobacteria* phage SfI | YP_009147459.1 | 100 | 100 |
| x | 31188 | 31562 | + | 375 | tail protein | *Burkholderia cenocepacia* | WP_060263169.1 | 100 | 100 |
| x | 32290 | 33714 | + | 1425 | multidrug DMT transporter | *Burkholderia cenocepacia* | WP_060263171.1 | 100 | 100 |
| x | 36429 | 36992 | + | 564 | base plate assembly | *Burkholderia cenocepacia* | WP_060263250.1 | 90 | 41 |
| 17 | 36996 | 37442 | + | 447 | tail protein | *Escherichia* virus Mu | NP_050650.1 | 97 | 43 |
| x | 37444 | 38607 | + | 1164 | baseplate protein | Burkholderia cenocepacia | WP_060263175.1 | 100 | 100 |
| x | 38893 | 39255 | + | 363 | tail protein | Burkholderia cenocepacia | WP_059486317.1 | 100 | 71 |
| x | 42722 | 43294 | + | 573 | lysozyme | Burkholderia cenocepacia | WP_060263180.1 | 98 | 100 |
| x | 44925 | 45524 | + | 600 | lipase | Burkholderia cenocepacia | WP_060263184.1 | 100 | 100 |
| 18 | 47602 | 48057 | - | 456 | hypothetical protein | *Burkholderia* phage KS9 | YP_003090204.1 | 98 | 44 |
| 19 | 48138 | 48818 | + | 681 | hypothetical protein | *Burkholderia* virus phiE125 | NP_536389.1 | 98 | 73 |

| **Terminators** | | | |
| --- | --- | --- | --- |
| **Strand** | **Start** | **End** | **Sequence** |
| + | 74 | 93 | CGCCGCTTCCGACAGCGGCG |
| - | 8010 | 8033 | GCGGGCGTCCTTGGTGGCGCCCGC |
| - | 10283 | 10299 | GCCCGCCGTGTGCGGGC |
| + | 19350 | 19375 | GCCTGCCGATCGAGAGACCGGCGGGC |
| + | 19431 | 19448 | CGCCCGGTTCGCCGGGCG |
| - | 21282 | 21301 | TCAGCCGCGGCGGCGGCTGA |
| + | 27153 | 27171 | GCCCCGCCTCGAGCGGGGC |
| + | 31143 | 34164 | CGCCGCCCTTAACCGGGCGGCG |
| + | 42302 | 42317 | GCCGCCTTCGGGCGGC |
| + | 43332 | 43349 | GCCCGGCCGCGCGCCGGG |
