# Supplementary material for: Burkholderia cenocepacia Prophages—Prevalence, Chromosome Location and Major Genes Involved
Source: Viruses. 2018 May 31;10(6):297. doi: 10.3390/v10060297 (PMC6024312; doi:10.3390/v10060297)
Supplement: Supplementary file 1 [file viruses-10-00297-s001.zip › viruses-297954-r2-supplementary OK/Supplementary data/Region Characteristics Cards/Supplementary_data_16_RC_VC7848_chr1_2.docx]

| **Region characteristics** | | | |
| --- | --- | --- | --- |
| Phage name: | VC7848_chr1_2 | | |
| Size (nt): | 38,294 | | |
| Type: | Prophage | | |
| Taxonomical affiliation (homology based): | Order: *Caudovirales*  Family: *Myoviridae*  Genus: *Peduovirinae* | | |
| Number of annotated open reading frames (ORF): | 53 | | |
| Number of annotated regulatory sequences: | Terminators: | 2 | |
|  | Promoters: | 0 | |
|  | tRNA: | 0 | |
| Derivation: | Host: | | *Burkholderia cenocepacia* VC7848  chromosome 1 |
|  | Sequence origin (database) | | NCBI |
|  | Accession number/version: | | NZ_CP019668.1 |
|  | Localization in genome: | | 6353033…6391326 |
|  | Additional information: | | - |
| Additional information: | -potential cos sites were found  - lytic cassette was found in positions #30-#33 (24356..26331)  - holds high homology to *Burkholderia phage* vB_BceM_AP3 | | |

| **Annotation** | | | | | |
| --- | --- | --- | --- | --- | --- |
| **#** | **Strand** | **Start** | **End** | **Length (nt)** | **Product** |
| x | x | 1 | 46 | 46 | attL |
| 1 | - | 111 | 1187 | 1077 | integrase |
| 2 | - | 2076 | 4868 | 2793 | hypothetical protein |
| 3 | - | 4871 | 5119 | 249 | hypothetical protein |
| 4 | - | 5116 | 5478 | 363 | hypothetical protein |
| 5 | - | 5482 | 5829 | 348 | hypothetical protein |
| 6 | - | 5834 | 6028 | 195 | hypothetical protein |
| 7 | - | 6072 | 6266 | 195 | hypothetical protein |
| 8 | - | 6271 | 6465 | 195 | hypothetical protein |
| 9 | - | 6554 | 6802 | 249 | transcriptional regulator |
| x | + | 6848 | 7039 | 192 | hypothetical protein |
| 10 | - | 7050 | 7241 | 192 | hypothetical protein |
| 11 | - | 7258 | 7443 | 186 | hypothetical protein |
| 12 | + | 7603 | 8052 | 450 | hypothetical protein |
| x | - | 8043 | 8423 | 381 | LysR family transcriptional regulator |
| x | + | 8597 | 8887 | 291 | hypothetical protein |
| 13 | - | 8890 | 10041 | 1152 | late control geneD protein |
| 14 | - | 10038 | 10466 | 429 | P2 GpU family protein |
| 15 | - | 10480 | 13731 | 3252 | hypothetical protein |
| 16 | - | 13728 | 13847 | 120 | P2 GpE family protein |
| 17 | - | 13847 | 14158 | 312 | tail protein |
| 18 | - | 14191 | 14700 | 510 | major tail tube protein |
| 19 | - | 14730 | 15902 | 1173 | tail sheath protein |
| 20 | - | 16014 | 16763 | 750 | DNA methylase N-4 |
| 21 | - | 17073 | 17411 | 339 | hypothetical protein |
| 22 | - | 17434 | 17868 | 435 | hypothetical protein |
| 23 | - | 17865 | 20882 | 3018 | phage tail fiber protein |
| 24 | - | 20888 | 21439 | 552 | tail protein |
| 25 | - | 21432 | 22337 | 906 | baseplate assembly protein |
| 26 | - | 22334 | 22699 | 366 | baseplate assembly protein |
| 27 | - | 22696 | 23400 | 705 | basteplate assembly protein |
| 28 | - | 23500 | 23949 | 450 | virion morphogenesis protein |
| 29 | - | 23949 | 24359 | 411 | tail completion protein (R) |
| 30 | - | 24356 | 24874 | 519 | i-spanin |
| 31 | - | 24844 | 25644 | 801 | endolysin |
| 32 | - | 25637 | 25957 | 321 | holin |
| 33 | - | 25957 | 26331 | 375 | antiholin |
| 34 | - | 26334 | 26546 | 213 | tail protein |
| 35 | - | 26546 | 26782 | 237 | hypothetical protein |
| 36 | - | 26782 | 27264 | 483 | head completion protein |
| 37 | - | 27369 | 28055 | 687 | small terminase subunit |
| 38 | - | 28052 | 29071 | 1020 | major capsid protein |
| 39 | - | 29108 | 29932 | 825 | capsid scaffolding protein |
| 40 | + | 30077 | 31840 | 1764 | terminase ATPase subunit (P) |
| 41 | + | 31956 | 32882 | 927 | portal vertex protein |
| 42 | - | 32910 | 33626 | 717 | hypothetical protein |
| 43 | + | 33721 | 34002 | 282 | hypothetical protein |
| 44 | + | 34051 | 34305 | 255 | cytotoxic repressor of toxin-antitoxin stability system |
| 45 | + | 34289 | 34615 | 327 | DNA-binding protein |
| 46 | - | 35008 | 36123 | 1116 | hypothetical protein |
| 47 | - | 36129 | 36899 | 771 | hypothetical protein |
| 48 | - | 36896 | 37432 | 537 | hypothetical protein |
| x | - | 37649 | 37966 | 318 | hypothetical protein |
| 49 |  | 38295 | 38340 | 46 | attR |

| **Terminators** | | | |
| --- | --- | --- | --- |
| **Strand** | **Start** | **End** | **Sequence** |
| - | 34634 | 34665 | GCCGCCGGCACCGAAGTGCCTGGCGGCTTTTT |
| + | 34639 | 34672 | GCCGCCAGGCACTTCGGTGCCGGCGGCTTTTTTT |
