# Supplementary material for: Burkholderia cenocepacia Prophages—Prevalence, Chromosome Location and Major Genes Involved
Source: Viruses. 2018 May 31;10(6):297. doi: 10.3390/v10060297 (PMC6024312; doi:10.3390/v10060297)
Supplement: Supplementary file 1 [file viruses-10-00297-s001.zip › viruses-297954-r2-supplementary OK/Supplementary data/Region Characteristics Cards/Supplementary_data_17_RC_VC12308_chr1_1.docx]

| **Region characteristics** | | | |
| --- | --- | --- | --- |
| Phage name: | VC12308_chr1_1 | | |
| Size (nt): | 22,704 | | |
| Type: | Prophage | | |
| Taxonomical affiliation (homology based): | Order: *Caudovirales*  Family: *Myoviridae*  Genus: *Peduovirinae* | | |
| Number of annotated open reading frames (ORF): | 29 | | |
| Number of annotated regulatory sequences: | Terminators: | 0 | |
|  | Promoters: | 0 | |
|  | tRNA: | 0 | |
| Derivation: | Host: | | *Burkholderia cenocepacia* VC12308, chromosome 1 |
|  | Sequence origin (database) | | NCBI |
|  | Accession number/version: | | NZ_CP019674.1 |
|  | Localization in genome: | | 70339.. 93042 |
|  | Additional information: | | - |
| Additional information: | - potential cos-sites found  - region holds homology to *Burkholderia phage* BEK  - the genome lacks crucial proteins – portal protein and teminase subunits. There is strong possibility that it is either artifact region or satellite phage. It was considered functional due to potential cos sites. | | |

| **Annotation** | | | | | |
| --- | --- | --- | --- | --- | --- |
| **#** | **Strand** | **Start** | **End** | **Length (nt)** | **Product** |
| 1 | + | 1 | 31 | 31 | attL |
| 2 | - | 100 | 1176 | 1077 | integrase |
| x | + | 1179 | 1451 | 273 | hypothetical protein |
| 3 | - | 1871 | 4663 | 2793 | zinc finger CHC2-family protein |
| 4 | - | 4669 | 4926 | 258 | hypothetical protein |
| 5 | - | 5054 | 5239 | 186 | ogr/Delta-like zinc finger family protein |
| 6 | - | 5484 | 5657 | 174 | hypothetical protein |
| 7 | + | 5790 | 6281 | 492 | repressor |
| 8 | + | 6571 | 6894 | 324 | hypothetical protein |
| 9 | - | 7082 | 8140 | 1059 | late control gene D protein |
| 10 | - | 8137 | 8568 | 432 | fels-2 prophage protein |
| 11 | - | 8591 | 11161 | 2571 | tail tape measure protein (T) |
| 12 | - | 11177 | 11290 | 114 | hypothetical protein |
| 13 | - | 11299 | 11670 | 372 | tail protein E |
| 14 | - | 11747 | 12256 | 510 | tail tube protein (FII) |
| 15 | - | 12285 | 13457 | 1173 | tail sheath protein |
| 16 | - | 13512 | 14264 | 753 | tail fiber assembly protein |
| 17 | - | 14280 | 16607 | 2328 | tail collar protein |
| 18 | - | 16614 | 17156 | 543 | tail protein |
| 19 | - | 17149 | 18063 | 915 | baseplate assembly protein (J) |
| 20 | - | 18060 | 18422 | 363 | baseplate assembly protein |
| 21 | - | 18419 | 19066 | 648 | baseplate assembly protein |
| 22 | - | 19614 | 20063 | 450 | tail completion protein |
| 23 | - | 20180 | 20620 | 441 | Rz |
| 24 | - | 20617 | 21474 | 858 | peptidoglycan binding protein |
| 25 | - | 21471 | 21737 | 267 | hypothetical protein |
| 26 | - | 21739 | 22083 | 345 | hypothetical protein |
| 27 | - | 22100 | 22306 | 207 | tail protein |
| 28 | + | 22705 | 22735 | 31 | attR |
