# Supplementary material for: Burkholderia cenocepacia Prophages—Prevalence, Chromosome Location and Major Genes Involved
Source: Viruses. 2018 May 31;10(6):297. doi: 10.3390/v10060297 (PMC6024312; doi:10.3390/v10060297)
Supplement: Supplementary file 1 [file viruses-10-00297-s001.zip › viruses-297954-r2-supplementary OK/Supplementary data/Region Characteristics Cards/Supplementary_data_2_RC_895_chr1_2.docx]

| **Region characteristics** | | | |
| --- | --- | --- | --- |
| Phage name: | 895_chr1_2 | | |
| Size (nt): | 38627 | | |
| Type: | Artifact region | | |
| Taxonomical affiliation (homology based): | - | | |
| Number of annotated open reading frames (ORF): | 19 | | |
| Number of annotated regulatory sequences: | Terminators: | - | |
|  | Promoters: | - | |
|  | tRNA: | - | |
| Deriviation: | Host: | | *Burkholderia cenocepacia* 895  chromosome 1 |
|  | Sequence origin (database) | | NCBI |
|  | Accession number/version: | | NZ_CP015036.1 |
|  | Localization in genome: | | 3737465..3776092 |
|  | Additional information: | | Even though Phaster recognize region as complete phage, annotation shows that it is probably non-functional virus. Genes found in this region, often show homology to phages from various taxonomical groups and specific to hosts other than *Burkholderia.* |
| Additional informations: | - Region contains sequences which might have served as *cos* sites  - almost 70% of the genes found in this region are homologues of transposase  - of the genes that were found in region:  a) 19 genes show homology with known phage genes  b) 25 genes with homology to bacterial genes (not mentioned in annotation table)  - homologue of gene 19 derives from viruses of *Ostreococcus* algae species | | |

| **Annotation** | | | | | | | | | |
| --- | --- | --- | --- | --- | --- | --- | --- | --- | --- |
| **#** | **Strand** | **Start** | **End** | **Length (nt)** | **Product** | **Homology** | | | |
|  |  |  |  |  |  | Phage name | Phage name | Phage name | Phage name |
| 1 | - | 141 | 1709 | 1569 | Hypothetical protein | *Streptococcus* phage phi-m46.1 | CAR95374.1 | 99 | 28 |
| 2 | + | 3956 | 4348 | 393 | DNA invertase | *Escherichia phage* pro483 | YP_009211932.1 | 76 | 36 |
| 3 | + | 4673 | 5020 | 348 | transposase | Stx2-converting phage 1717 | YP_002274237.1 | 93 | 64 |
| 4 | + | 5072 | 6616 | 1545 | transposase, IS66 family | Stx2-converting phage Stx2a_1447 | BAT32436.1 | 98 | 48 |
| 5 | + | 7276 | 7629 | 354 | DNA invertase | *Enterobacteria* phage mEp237 | YP_007111399.1 | 72 | 35 |
| 6 | - | 8938 | 9804 | 867 | IS21 transposition protein | *Enterobacteria* phage fiAA91-ss | YP_008766949.1 | 79 | 44 |
| 7 | - | 9801 | 11057 | 1257 | IS21 transposase | *Enterobacteria* phage fiAA91-ss | YP_008766948.1 | 95 | 33 |
| 8 | - | 11865 | 12653 | 789 | IS21 transposition protein | *Enterobacteria* phage fiAA91-ss | YP_008766949.1 | 89 | 37 |
| 9 | - | 12658 | 14190 | 1533 | transposase IstA, IS21 family | *Acidithiobacillus* phage AcaML1 | AFU62914.1 | 71 | 30 |
| 10 | - | 15303 | 16043 | 741 | putative transposase OrfB | *Enterobacteria* phage Sf6 | NP_958229.1 | 96 | 58 |
| 11 | - | 16157 | 16489 | 333 | gene 56 protein | *Enterobacteria* phage Sf6 | NP_958230.1 | 90 | 47 |
| 12 | - | 17242 | 17991 | 750 | transposition helper protein IstB, IS21 family | *Acidithiobacillus* phage AcaML1 | AFU62915.1 | 97 | 61 |
| 13 | - | 18008 | 19549 | 1542 | transposase IstA, IS21 family | *Acidithiobacillus* phage AcaML1 | AFU62914.1 | 100 | 55 |
| 14 | - | 21276 | 22820 | 1542 | transposase, IS66 family | Stx2-converting phage Stx2a_1447 | BAT32436.1 | 98 | 48 |
| 15 | - | 22872 | 23219 | 348 | transposase | Stx2-converting phage 1717 | YP_002274237.1 | 93 | 64 |
| 16 | - | 23216 | 23647 | 432 | truncated transposase | Stx2-converting phage 1717 | YP_002274236.1 | 96 | 36 |
| 17 | - | 27507 | 28535 | 1032 | putative integrase | *Pseudomonas* phage PAJU2 | YP_002284365.1 | 78 | 43 |
| 18 | - | 28789 | 29757 | 969 | shufflon-specific DNA recombinase | *Ralstonia* phage RS-PII-1 | APU00288.1 | 99 | 41 |
| 19 | + | 30158 | 30907 | 750 | hypothetical protein OtV6_169c | *Ostreococcus* *tauri* virus RT-2011 | AFC35077.1 | 77 | 30 |
