# Supplementary material for: Burkholderia cenocepacia Prophages—Prevalence, Chromosome Location and Major Genes Involved
Source: Viruses. 2018 May 31;10(6):297. doi: 10.3390/v10060297 (PMC6024312; doi:10.3390/v10060297)
Supplement: Supplementary file 1 [file viruses-10-00297-s001.zip › viruses-297954-r2-supplementary OK/Supplementary data/Region Characteristics Cards/Supplementary_data_3_RC_895_chr1_3.docx]

| **Region characteristics** | | | |
| --- | --- | --- | --- |
| Phage name: | 895_chr1_3 | | |
| Size (nt): | 43300 | | |
| Type: | Artifact region | | |
| Taxonomical affiliation (homology based): | - | | |
| Number of annotated open reading frames (ORF): | 60 | | |
| Number of annotated regulatory sequences: | Terminators: | - | |
|  | Promoters: | - | |
|  | tRNA: | - | |
| Derivation: | Host: | | *Burkholderia cenocepacia* 895  chromosom 1 |
|  | Sequence origin (database) | | NCBI |
|  | Accession number/version: | | NZ_CP015036.1 |
|  | Localization in genome: | | 4023532..4066832 |
|  | Additional information: | | Even though Phaster recognize region as complete phage, annotation shows that it is probably non-functional virus. Genes found in this region, often show homology to phages from various taxonomical groups and specific to hosts other than *Burkholderia.* |
| Additional information: | - potential *cos* sites present  - of the genes that were found in region:  a) 25 genes show homology with known phage genes  b) 5 genes are distinctive for phages, although with no homology to viral sequences in the database (green)  c) 28 genes with homology to bacterial genes (not mentioned in annotation table) | | |

| **Annotation** | | | | | | | | | |
| --- | --- | --- | --- | --- | --- | --- | --- | --- | --- |
| **#** | **Strand** | **Start** | **End** | **Length (nt)** | **Product** | **Homology** | | | |
|  |  |  |  |  |  | Phage name | A/N | QC % | Ident% |
| 1 | - | 7730 | 8296 | 567 | putative endolysin | *Pseudomonas* phage phiPSA1 | YP_009043563.1 | 89 | 44 |
| 2 | - | 8299 | 8685 | 387 | lysozyme | *Burkholderia* *cenocepacia* | WP_062910680.1 | 100 | 100 |
| 3 | - | 8758 | 10656 | 1899 | tail fiber protein | *Burkholderia* virus Bcep781 | NP_705672.2 | 78 | 29 |
| 4 | - | 10696 | 11241 | 546 | hypothetical protein | *Ralstonia* phage RSL1 | YP_001950073.1 | 88 | 35 |
| 5 | - | 12185 | 12826 | 642 | tail protein | *Burkholderia cenocepacia* | WP_080466890.1 | 100 | 100 |
| 6 | - | 12827 | 13990 | 1164 | baseplate J like protein | *Escherichia* virus Mu | NP_050651.1 | 99 | 33 |
| 7 | - | 15107 | 16261 | 1155 | tail protein | *Shigella* phage SfIV | YP_008766879.1 | 96 | 32 |
| 8 | - | 16258 | 17658 | 1401 | tail protein | *Salmonella* phage 118970_sal3 | YP_009324803.1 | 93 | 26 |
| 9 | - | 20102 | 20473 | 372 | tail protein | *Burkholderia* *cenocepacia* | WP_062910690.1 | 100 | 100 |
| 10 | - | 20535 | 22019 | 1485 | sheath protein | *Enterobacteria* phage phiP27 | NP_543096.1 | 99 | 42 |
| 11 | - | 22226 | 22789 | 564 | hypothetical protein | *Burkholderia* virus phi1026b | NP_945103.1 | 98 | 48 |
| 12 | - | 23111 | 23377 | 267 | major capsid protein | *Burkholderia* phage AH2 | YP_006561146.1 | 94 | 33 |
| 13 | - | 23377 | 24447 | 1071 | capsid protein | *Burkholderia cenocepacia* | WP_062910696.1 | 99 | 100 |
| 14 | - | 25878 | 26768 | 891 | peptidase | *Burkholderia* virus phi6442 | YP_001111084.1 | 87 | 41 |
| 15 | - | 26768 | 28402 | 1635 | portal protein | *Salmonella* phage SPN19 | YP_006990307.1 | 89 | 35 |
| 16 | - | 28402 | 28650 | 249 | hypothetical protein | *Burkholderia cepacia* | WP_017918055.1 | 100 | 100 |
| 17 | - | 28661 | 30742 | 2082 | terminase large subunit | *Acidithiobacillus* phage AcaML1 | AFU62879.1 | 84 | 38 |
| 18 | - | 31588 | 32208 | 621 | minor tail protein | *Rhodobacter* phage RcCronus | AKU43333.1 | 99 | 38 |
| 19 | - | 33156 | 33416 | 261 | hypothetical protein | *Burkholderia* virus Bcep22 | NP_944247.1 | 86 | 54 |
| 20 | - | 33416 | 33655 | 240 | hypothetical protein | *Burkholderia* virus phiE125 | NP_536422.1 | 100 | 48 |
| 21 | - | 33652 | 34140 | 489 | hypothetical protein | *Pseudomonas* phage PMG1 | YP_005098280.1 | 99 | 39 |
| 22 | - | 34137 | 34520 | 384 | hypothetical protein | *Burkholderia* virus phi1026b | NP_945106.1 | 86 | 76 |
| 23 | - | 36101 | 36277 | 177 | hypothetical protein | *Burkholderia* phage Bcep176 | YP_355344.1 | 100 | 78 |
| 24 | + | 39419 | 40081 | 663 | cI repressor protein | *Pseudomonas* phage JBD44 | YP_009275564.1 | 96 | 30 |
| 25 | + | 41976 | 42266 | 291 | hypothetical protein | *Burkholderia* virus Bcep781 | YP_022742.1 | 94 | 63 |
