# Supplementary material for: Burkholderia cenocepacia Prophages—Prevalence, Chromosome Location and Major Genes Involved
Source: Viruses. 2018 May 31;10(6):297. doi: 10.3390/v10060297 (PMC6024312; doi:10.3390/v10060297)
Supplement: Supplementary file 1 [file viruses-10-00297-s001.zip › viruses-297954-r2-supplementary OK/Supplementary data/Region Characteristics Cards/Supplementary_data_4_RC_895_chr1_7.docx]

| **Region characteristics** | | | |
| --- | --- | --- | --- |
| Phage name: | 895_chr1_7 | | |
| Size (nt): | 26721 | | |
| Type: | Prophage | | |
| Taxonomical affiliation (homology based): | Order: *Caudovirales*  Family: *Myoviridae* | | |
| Number of annotated open reading frames (ORF): | 29 | | |
| Number of annotated regulatory sequences: | Terminators: | 0 | |
|  | Promoters: | 0 | |
|  | tRNA: | 0 | |
| Derivation: | Host: | | *Burkholderia cenocepacia* 895  chromosome 1 |
|  | Sequence origin (database) | | NCBI |
|  | Accession number/version: | | NZ_CP015036.1 |
|  | Localization in genome: | | 5741212..5767932 |
|  | Additional information: | | Probably non-active phage. Even though homology to viral database is high and presence of *cos* site, mosaic type of build of the genome and non-typical location of the integrase, may suggest of phage inactivity or artifactual character of the region. |
| Additional information: | - potential lytic cassette in position potencjalna kaseta lityczna na pozycji 4545..6448  - integrase located downstream to *cos* site  - integrase homologous to virus specific to other bacteria specie  - mosaic genome construction  - no regulatory sequences found in the genome | | |

| **Annotation** | | | | | |
| --- | --- | --- | --- | --- | --- |
| **#** | **Strand** | **Start** | **End** | **Length (nt)** | **Product** |
| 1 | + | 1 | 12 | 12 | cosL; left cohesive end |
| 2 | - | 1849 | 3468 | 1620 | hypothetical protein |
| 3 | + | 4032 | 4238 | 207 | Tail protein X |
| 4 | + | 4255 | 4599 | 345 | putative holin |
| 5 | + | 4601 | 4867 | 267 | putative holin |
| 6 | + | 4864 | 5721 | 858 | hypothetical protein |
| 7 | + | 5718 | 6158 | 441 | Rz |
| 8 | + | 6275 | 6724 | 450 | Tail protein GpR |
| 9 | + | 7272 | 7919 | 648 | baseplate assembly (V family) protein |
| 10 | + | 7916 | 8278 | 363 | baseplate assembly protein (W) |
| 11 | + | 8275 | 9189 | 915 | baseplate assembly protein (J) |
| 12 | + | 9182 | 9724 | 543 | tail protein I |
| 13 | + | 9731 | 12301 | 2571 | tail fiber protein |
| 14 | + | 12318 | 13007 | 690 | tail fiber assembly protein |
| 15 | + | 13062 | 14234 | 1173 | major tail sheath protein |
| 16 | + | 14269 | 14772 | 504 | major tail tube protein |
| 17 | + | 14788 | 15219 | 432 | tail protein (E) |
| 18 | + | 15228 | 15341 | 114 | tail protein GpE family |
| 19 | + | 15357 | 17927 | 2571 | tail tape measure protein (T) |
| 20 | + | 17950 | 18381 | 432 | fels-2 |
| 21 | + | 18378 | 19448 | 1071 | bacteriophage late control gene D protein |
| 22 | - | 19636 | 19959 | 324 | hypothetical protein |
| 23 | - | 20250 | 20741 | 492 | hypothetical protein |
| 24 | + | 20874 | 21047 | 174 | hypothetical protein |
| 25 | + | 21062 | 21241 | 180 | hypothetical protein |
| 26 | + | 21229 | 21477 | 249 | ogr/Delta-like zinc finger family protein |
| 27 | + | 21605 | 21862 | 258 | hypothetical protein |
| 28 | + | 21868 | 24660 | 2793 | zinc finger CHC2-family protein |
| 29 | + | 25117 | 25128 | 12 | cosR; right cohesive end |
| 30 | - | 25080 | 25352 | 273 | hypothetical protein |
| 31 | + | 25355 | 26431 | 1077 | integrase |

| **Terminators** | | | |
| --- | --- | --- | --- |
| **Strand** | **Start** | **End** | **Sequence** |
| - | - | - | --- |
