# Supplementary material for: Burkholderia cenocepacia Prophages—Prevalence, Chromosome Location and Major Genes Involved
Source: Viruses. 2018 May 31;10(6):297. doi: 10.3390/v10060297 (PMC6024312; doi:10.3390/v10060297)
Supplement: Supplementary file 1 [file viruses-10-00297-s001.zip › viruses-297954-r2-supplementary OK/Supplementary data/Region Characteristics Cards/Supplementary_data_5_RC_895_chr1_9.docx]

| **Region characteristics** | | | |
| --- | --- | --- | --- |
| Phage name: | 895_chr1_9 | | |
| Size (nt): | 40988 | | |
| Type: | Prophage | | |
| Taxonomical affiliation (homology based): | Order: *Caudovirales*  Family: *Siphoviridae* | | |
| Number of annotated open reading frames (ORF): | 50 | | |
| Number of annotated regulatory sequences: | Terminators: | 8 | |
|  | Promoters: | 0 | |
|  | tRNA: | 0 | |
| Derivation: | Host: | | *Burkholderia cenocepacia* 895  chromosome 1 |
|  | Sequence origin (database) | | NCBI |
|  | Accession number/version: | | NZ_CP015036.1 |
|  | Localization in genome: | | 6823839..6864827 |
|  | Additional information: | | - |
| Additional information: | - start position of the phage genome has been altered in comparison to Phaster (6831265), due to the fact that cos sites and integrase genes have been omitted by it  - end position of the phage genome has been altered in comparison to Phaster (6868036), due to lack of homology between the proteins in the region and viral database  - *cos* sites have been found  - potential lytic cassette was found in position 35923..37138  - 2 genes are distinctive for phages, although with no homology to viral sequences in the database (green)  - 9 genes with homology to bacterial genes have been found (blue) | | |

| **Annotation** | | | | | |
| --- | --- | --- | --- | --- | --- |
| **#** | **Strand** | **Start** | **End** | **Length (nt)** | **Product** |
| 1 | + | 1 | 16 | 16 | left cos-site |
| 2 | + | 184 | 1194 | 1011 | integrase |
| 3 | - | 1191 | 1496 | 306 | hypothetical protein |
| 4 | - | 1496 | 2350 | 855 | hypothetical protein |
| 5 | - | 2467 | 3207 | 741 | dinucleotide-utilizing protein |
| 6 | - | 3204 | 3584 | 381 | hypothetical protein |
| 7 | - | 3581 | 4384 | 804 | hypothetical protein |
| 8 | - | 4389 | 5099 | 711 | hypothetical protein |
| 9 | - | 5096 | 6175 | 1080 | hypothetical protein |
| 10 | - | 6354 | 6836 | 483 | hypothetical protein |
| 11 | - | 6851 | 7405 | 555 | hypothetical protein |
| 12 | - | 7721 | 7927 | 207 | hypothetical protein |
| 13 | - | 7935 | 8060 | 126 | hypothetical protein |
| 14 | - | 8057 | 8281 | 225 | hypothetical protein |
| 15 | - | 8290 | 8430 | 141 | hypothetical protein |
| 16 | - | 8817 | 9281 | 465 | transcriptional regulator |
| 17 | + | 9351 | 9632 | 282 | hypothetical protein |
| 18 | + | 9758 | 10369 | 612 | hypothetical protein |
| 19 | + | 10396 | 11763 | 1368 | helicase |
| 20 | + | 11802 | 12773 | 972 | replication protein |
| 21 | + | 12786 | 13184 | 399 | hypothetical protein |
| 22 | + | 13344 | 13577 | 234 | hypothetical protein |
| 23 | + | 14548 | 15084 | 537 | terminase, small subunit |
| 24 | + | 15129 | 16793 | 1665 | terminase, large subunit |
| 25 | + | 16790 | 18079 | 1290 | portal protein |
| 26 | + | 18045 | 18860 | 816 | protease |
| 27 | + | 18929 | 20194 | 1266 | major capsid protein |
| 28 | + | 20194 | 20829 | 636 | hypothetical protein |
| 29 | + | 20840 | 21166 | 327 | head-tail adaptor protein |
| 30 | + | 21159 | 21581 | 423 | hypothetical protein |
| 31 | + | 21578 | 21922 | 345 | hypothetical protein |
| 32 | + | 21983 | 22447 | 465 | major tail subunit |
| 33 | + | 22476 | 22943 | 468 | tail assembly chaperone |
| 34 | + | 22991 | 23218 | 228 | minor tail protein |
| 35 | + | 23232 | 27332 | 4101 | tail length tape measure protein |
| 36 | + | 27332 | 27670 | 339 | minor tail protein |
| 37 | + | 27934 | 28893 | 960 | hypothetical protein |
| 38 | + | 28896 | 29579 | 684 | minor tail protein |
| 39 | + | 29629 | 30381 | 753 | tail component protein |
| 40 | + | 30938 | 34843 | 3906 | tail tip fiber protein |
| 41 | + | 35151 | 35867 | 717 | hypothetical protein |
| 42 | + | 35923 | 36207 | 285 | holin |
| 43 | + | 36210 | 36659 | 450 | endolysin |
| 44 | + | 36656 | 37138 | 483 | Rz |
| 45 | + | 37277 | 38068 | 792 | DNA adenine methylase |
| 46 | + | 38324 | 39067 | 744 | hypothetical protein |
| 47 | - | 39169 | 39594 | 426 | hypothetical protein |
| 48 | - | 39653 | 40120 | 468 | hypothetical protein |
| 49 | + | 40197 | 40901 | 705 | hypothetical protein |
| 50 | + | 40974 | 40989 | 16 | right cos-site |

| **Terminators** | | | |
| --- | --- | --- | --- |
| **Strand** | **Start** | **End** | **Sequence** |
| + | 2410 | 2436 | GCCCGCTCGCGTTTCGCGTGAGCGGGC |
| + | 13275 | 13298 | GCCTCGACTGCGAAAGCGTCGGGG |
| + | 18883 | 18902 | GGGCTGCCCTCGGGTGGCCC |
| + | 21942 | 21959 | GGCCCGCCTCGAGCGGGC |
| + | 29606 | 29621 | CCCGCCAAACGGCGGG |
| + | 35869 | 35888 | GGGCCGCCAACTTGGCGGCC |
| + | 37145 | 37183 | GGCGACCGAGCGGCGTGCGGTAACACGCCCCTCGGTCGC |
| + | 37074 | 38092 | GCCCGCGCTTGCCGCGGGC |
