# Supplementary material for: Burkholderia cenocepacia Prophages—Prevalence, Chromosome Location and Major Genes Involved
Source: Viruses. 2018 May 31;10(6):297. doi: 10.3390/v10060297 (PMC6024312; doi:10.3390/v10060297)
Supplement: Supplementary file 1 [file viruses-10-00297-s001.zip › viruses-297954-r2-supplementary OK/Supplementary data/Region Characteristics Cards/Supplementary_data_6_RC_895_chr2_1.docx]

| **Region characteristics** | | | |
| --- | --- | --- | --- |
| Phage name: | 895_chr2_1 | | |
| Size (nt): | 37652 | | |
| Type: | Prophage | | |
| Taxonomical affiliation (homology based): | Order: *Caudovirales*  Family: *Myoviridae* | | |
| Number of annotated open reading frames (ORF): | 48 | | |
| Number of annotated regulatory sequences: | Terminators: | 3 | |
|  | Promoters: | 0 | |
|  | tRNA: | 0 | |
| Derivation: | Host: | | *Burkholderia cenocepacia* 895  chromosome 2 |
|  | Sequence origin (database) | | NCBI |
|  | Accession number/version: | | CP015037.1 |
|  | Localization in genome: | | 826395..864047 |
|  | Additional information: | | - |
| Additional information: | - at location #12-13 gene has been disrupted. Stop codon has been itroduced, which cuts protein ParB, between genes 12 and 13  - 6 genes with homology to bacterial genes have been found (blue) | | |

| **Annotation** | | | | | |
| --- | --- | --- | --- | --- | --- |
| **#** | **Strand** | **Start** | **End** | **Length (nt)** | **Product** |
| 1 | + | 1 | 1251 | 1251 | IS256 family transposase |
| 2 | - | 1879 | 2217 | 339 | hypothetical protein |
| 3 | + | 2589 | 3485 | 897 | hypothetical protein |
| 4 | + | 3482 | 3982 | 501 | hypothetical protein |
| 5 | + | 4013 | 4192 | 180 | hypothetical protein |
| 6 | + | 4189 | 4419 | 231 | hypothetical protein |
| 7 | + | 4416 | 5321 | 906 | DNA methyltransferase |
| 8 | + | 5323 | 5538 | 216 | hypothetical protein |
| 9 | + | 5708 | 6691 | 984 | PAPS reductase/sulfotransferase |
| 10 | + | 6703 | 8604 | 1902 | replication A protein |
| 11 | + | 8601 | 8879 | 279 | transcriptional regulator, Ogr/Delta |
| 12 | + | 9159 | 9491 | 333 | ParB-like partition protein [Burkholderia phage ST79] |
| 13 | + | 9502 | 10086 | 585 | hypothetical protein |
| 14 | + | 10083 | 10868 | 786 | hypothetical protein |
| 15 | + | 10865 | 11272 | 408 | hypothetical protein |
| 16 | - | 11483 | 11872 | 390 | HicB |
| 17 | - | 11869 | 12123 | 255 | HicA |
| 18 | - | 12547 | 13608 | 1062 | portal protein |
| 19 | - | 13608 | 15425 | 1818 | terminase ATPase subunit |
| 20 | + | 15580 | 16446 | 867 | capsid scaffolding protein |
| 21 | + | 16492 | 17529 | 1038 | major capsid protein |
| 22 | + | 17572 | 18237 | 666 | terminase endonuclease subunit |
| 23 | + | 18334 | 18834 | 501 | head completion/stabilization protein |
| 24 | + | 18834 | 19088 | 255 | hypothetical protein |
| 25 | + | 19085 | 19291 | 207 | tail protein X |
| 26 | + | 19299 | 19742 | 444 | hypothetical protein |
| 27 | + | 19730 | 20134 | 405 | hypothetical protein |
| 28 | + | 20170 | 20619 | 450 | peptidase |
| 29 | + | 20616 | 21056 | 441 | LysB |
| 30 | + | 21058 | 21186 | 129 | LysC |
| 31 | + | 21183 | 21659 | 477 | tail completion protein |
| 32 | + | 21659 | 22129 | 471 | virion morphogenesis protein |
| 33 | + | 22263 | 22979 | 717 | baseplate assembly protein V |
| 34 | + | 22979 | 23353 | 375 | baseplate assembly protein W |
| 35 | + | 23350 | 24258 | 909 | baseplate assembly protein J |
| 36 | + | 24248 | 24793 | 546 | tail protein I |
| 37 | + | 24790 | 28155 | 3366 | tail fiber protein |
| 38 | + | 28168 | 28578 | 411 | hypothetical protein |
| 39 | + | 28629 | 28961 | 333 | hypothetical protein |
| 40 | + | 29029 | 30216 | 1188 | major tail sheath protein |
| 41 | + | 30278 | 30781 | 504 | major tail tube protein |
| 42 | + | 30835 | 31140 | 306 | tail protein E |
| 43 | + | 31261 | 34179 | 2919 | tail protein E |
| 44 | + | 34187 | 34693 | 507 | tail protein U |
| 45 | + | 34698 | 35777 | 1080 | tail protein D |
| 46 | - | 35815 | 36177 | 363 | hypothetical protein |
| 47 | - | 36256 | 36993 | 738 | hypothetical protein |
| 48 | - | 37111 | 37653 | 543 | hypothetical protein |

| **Terminators** | | | |
| --- | --- | --- | --- |
| **Strand** | **Start** | **End** | **Sequence** |
| - | 11366 | 11382 | TGCCCGCGTTGCGGGCG |
| - | 35782 | 35799 | GCCCGCACTTGTGCGGGC |
| - | 35782 | 35799 | GCCCGCACAAGTGCGGGC |
