# Supplementary material for: Burkholderia cenocepacia Prophages—Prevalence, Chromosome Location and Major Genes Involved
Source: Viruses. 2018 May 31;10(6):297. doi: 10.3390/v10060297 (PMC6024312; doi:10.3390/v10060297)
Supplement: Supplementary file 1 [file viruses-10-00297-s001.zip › viruses-297954-r2-supplementary OK/Supplementary data/Region Characteristics Cards/Supplementary_data_7_RC_CR 318_chr1_1.docx]

| **Region characteristics** | | | |
| --- | --- | --- | --- |
| Phage name: | CR 318_chr1_1 | | |
| Size (nt): | 38,476 | | |
| Type: | Prophage | | |
| Taxonomical affiliation (homology based): | Order: *Caudovirales*  Family: *Myoviridae*  Genus: *Peduovirinae* | | |
| Number of annotated open reading frames (ORF): | 45 | | |
| Number of annotated regulatory sequences: | Terminators: | 2 | |
|  | Promoters: | 0 | |
|  | tRNA: | 0 | |
| Derivation: | Host: | | *Burkholderia cenocepacia* CR 318  chromosome 1 |
|  | Sequence origin (database) | | NCBI |
|  | Accession number/version: | | NZ_CP017238.1 |
|  | Localization in genome: | | 300609…339084 |
|  | Additional information: | | - |
| Additional information: | - prophage integrates in tRNA-Arg  - potential cohesive ends found  - bacteriophage homologous to temperate phage *Burkholderia* phage KL3 (GU911304.1)  - potential lytic cassette was found in position #28-#31 (23023..24884) | | |

| **Annotation** | | | | | |
| --- | --- | --- | --- | --- | --- |
| **#** | **Strand** | **Start** | **End** | **Length (nt)** | **Product** |
| x | x | 1 | 46 | 46 | attL |
| 1 | - | 111 | 1232 | 1122 | integrase |
| 2 | - | 1513 | 2124 | 612 | baseplate J family protein |
| 3 | - | 2121 | 4913 | 2793 | hypothetical protein |
| 4 | - | 4916 | 5182 | 267 | hypothetical protein |
| 5 | - | 5179 | 5541 | 363 | hypothetical protein |
| 6 | - | 5546 | 5740 | 195 | hypothetical protein |
| 7 | - | 5987 | 6199 | 213 | hypothetical protein |
| 8 | - | 6286 | 6471 | 186 | transcriptional activator (Org) |
| 9 | - | 6522 | 6725 | 204 | hypothetical protein |
| 10 | - | 6765 | 6962 | 198 | hypothetical protein |
| 11 | + | 7236 | 7763 | 528 | transcriptional regulator |
| 12 | + | 7935 | 8222 | 288 | hypothetical protein |
| 13 | - | 8269 | 9360 | 1092 | tail protein (D) |
| 14 | - | 9357 | 9815 | 459 | tail protein (U) |
| 15 | - | 9837 | 12998 | 3162 | tail tape measure protein (T) |
| 16 | - | 13123 | 13473 | 351 | tail protein Es |
| 17 | - | 13540 | 14049 | 510 | tail tube protein (FII) |
| 18 | - | 14065 | 15237 | 1173 | phage tail sheath protein |
| 19 | - | 15290 | 15913 | 624 | tail fiber assembly protein |
| 20 | - | 15931 | 18597 | 2667 | tail fiber protein |
| 21 | - | 18601 | 19143 | 543 | baseplate assembly protein (I) |
| 22 | - | 19148 | 20053 | 906 | baseplate assembly protein (J) |
| 23 | - | 20050 | 20412 | 363 | baseplate assembly protein (W) |
| 24 | - | 20409 | 21104 | 696 | baseplate assembly protein (V) |
| 25 | + | 21270 | 22046 | 777 | N-4/N-6 DNA methylase |
| 26 | - | 22026 | 22493 | 468 | tail completion protein (S) |
| 27 | - | 22493 | 22909 | 417 | tail completion protein (R) |
| 28 | - | 23023 | 23463 | 441 | Rz |
| 29 | - | 23460 | 24269 | 810 | endolysin |
| 30 | - | 24266 | 24538 | 273 | holin |
| 31 | - | 24540 | 24884 | 345 | antiholin |
| 32 | - | 24900 | 25109 | 210 | tail protein (X) |
| 33 | - | 25341 | 25820 | 480 | capsid completion protein (L) |
| 34 | - | 25920 | 26609 | 690 | terminase endonuclease subunit |
| 35 | - | 26606 | 27619 | 1014 | major capsid protein |
| 36 | - | 27654 | 28445 | 792 | capsid scaffolding protein |
| 37 | + | 28590 | 30359 | 1770 | terminase ATPase subunit (P) |
| 38 | + | 30356 | 31426 | 1071 | portal protein |
| 39 | + | 33412 | 33729 | 318 | hypothetical protein |
| 40 | - | 33774 | 34862 | 1089 | hypothetical protein |
| 41 | - | 34868 | 35572 | 705 | hypothetical protein |
| 42 | - | 35569 | 36078 | 510 | hypothetical protein |
| 43 | + | 36173 | 36949 | 777 | hypothetical protein |
| x | x | 38476 | 38521 | 46 | attR |

| **Terminators** | | | |
| --- | --- | --- | --- |
| **Strand** | **Start** | **End** | **Sequence** |
| + | 8249 | 8270 | GCCCGCCGCGTGCGGGCTTTTT |
| + | 31567 | 31599 | CCGGCCGCGGTCCCCCTGCGGCCGGTTTTTTTT |
