# Supplementary material for: Burkholderia cenocepacia Prophages—Prevalence, Chromosome Location and Major Genes Involved
Source: Viruses. 2018 May 31;10(6):297. doi: 10.3390/v10060297 (PMC6024312; doi:10.3390/v10060297)
Supplement: Supplementary file 1 [file viruses-10-00297-s001.zip › viruses-297954-r2-supplementary OK/Supplementary data/Region Characteristics Cards/Supplementary_data_8_RC_DDS 22E-1_chr1_2.docx]

| **Region characteristics** | | | |
| --- | --- | --- | --- |
| Phage name: | DDS 22E-1_chr1_2 | | |
| Size (nt): | 31,310 | | |
| Type: | Prophage | | |
| Taxonomical affiliation (homology based): | Order: *Caudovirales*  Family: *Siphoviridae* | | |
| Number of annotated open reading frames (ORF): | 47 | | |
| Number of annotated regulatory sequences: | Terminators: | 4 | |
|  | Promoters: | 0 | |
|  | tRNA: | 0 | |
| Derivation: | Host: | | *Burkholderia cenocepacia* DDS 22E-1  chromosome 1 |
|  | Sequence origin (database) | | NCBI |
|  | Accession number/version: | | NZ_CP007783.1 |
|  | Localization in genome: | | 1625635…1656945 |
|  | Additional information: | | - |
| Additional information: | - virus have mosaic construction  - prophage integrates in tRNA-Arg  - bacteriophage homologous to temperate phage *Burkholderia* phage vB_BceM_AP3 (KP966108.1)  - potential lytic cassette was found in position #28-#31 (24624...26599)  - HicB fimily protein was found (similar to bacterial TA-system component) at 9552…9157 (HicB)  - 15 of the genes found in bacteriophage don’t hold homology with virus genes database | | |

| **Annotation** | | | | | |
| --- | --- | --- | --- | --- | --- |
| **#** | **Strand** | **Start** | **End** | **Length (nt)** | **Product** |
| 1 | + | 1 | 333 | 333 | hypothetical protein |
| 2 | + | 795 | 1001 | 207 | helix-turn-helix family protein |
| 3 | + | 998 | 1147 | 150 | hypothetical protein |
| 4 | - | 1134 | 1319 | 186 | hypothetical protein |
| 5 | + | 1481 | 1780 | 300 | DNA binding protein |
| 6 | + | 2202 | 3209 | 1008 | DNA partitioning protein |
| 7 | + | 3297 | 3647 | 351 | hypothetical protein |
| 8 | + | 3685 | 4722 | 1038 | hypothetical protein |
| 9 | + | 4719 | 4895 | 177 | hypothetical protein |
| 10 | + | 4892 | 5215 | 324 | hypothetical protein |
| 11 | + | 5236 | 5775 | 540 | endonuclease |
| 12 | + | 5775 | 6107 | 333 | hypothetical protein |
| 13 | + | 6377 | 6547 | 171 | hypothetical protein |
| 14 | + | 6544 | 6882 | 339 | hypothetical protein |
| 15 | + | 7166 | 7588 | 423 | hypothetical protein |
| 16 | + | 7592 | 8608 | 1017 | hypothetical protein |
| 17 | - | 8826 | 9089 | 264 | hypothetical protein |
| 18 | - | 9157 | 9552 | 396 | HicB family protein |
| 19 | - | 9577 | 9759 | 183 | hypothetical protein |
| 20 | + | 9801 | 10157 | 357 | holin |
| 21 | + | 10322 | 10798 | 477 | terminase small subunit |
| 22 | + | 10802 | 12517 | 1716 | terminase large subunit |
| 23 | + | 12514 | 12699 | 186 | hypothetical protein |
| 24 | + | 12704 | 13963 | 1260 | hypothetical protein |
| 25 | + | 13960 | 14928 | 969 | peptidase |
| 26 | + | 15031 | 16338 | 1308 | major capsid head protein |
| 27 | + | 16398 | 16583 | 186 | hypothetical protein |
| 28 | + | 16589 | 17152 | 564 | hypothetical protein |
| 29 | + | 17152 | 17487 | 336 | hypothetical protein |
| 30 | + | 17477 | 17947 | 471 | hypothetical protein |
| 31 | + | 17954 | 18544 | 591 | hypothetical protein |
| 32 | + | 18571 | 18798 | 228 | hypothetical protein |
| 33 | + | 18795 | 20291 | 1497 | tail sheath protein |
| 34 | + | 20362 | 20709 | 348 | tail tube protein |
| 35 | + | 20706 | 20996 | 291 | tail protein E |
| 36 | + | 21130 | 23067 | 1938 | tail protein |
| 37 | + | 23075 | 24313 | 1239 | hypothetical protein |
| 38 | + | 24314 | 25429 | 1116 | tail protein P |
| 39 | + | 25426 | 26004 | 579 | hypothetical protein |
| 40 | + | 26001 | 26429 | 429 | hypothetical protein |
| 41 | + | 26401 | 27447 | 1047 | tail protein |
| 42 | + | 27438 | 28037 | 600 | hypothetical protein |
| 43 | + | 28041 | 29036 | 996 | tail collar domain protein |
| 44 | + | 29033 | 29716 | 684 | tail fiber assembly protein |
| 45 | + | 29817 | 30209 | 393 | endolysin |
| 46 | + | 30554 | 30823 | 270 | hypothetical protein |
| 47 | + | 30820 | 31311 | 492 | Rz |

| **Terminators** | | | |
| --- | --- | --- | --- |
| **Strand** | **Start** | **End** | **Sequence** |
| + | 8722 | 8750 | GCCCGCCCGGTTTGCCGTGCGGGCTTTTT |
| + | 14992 | 15017 | TGCCACCTTCGGGTGGCATTTTTTTT |
| + | 29739 | 29760 | GCCGCCTTCGGCGGCTTTTTTT |
| + | 29767 | 29793 | GGCCGCTTCTTGCGGCCaTTCGTTTTT |
