# Supplementary material for: Burkholderia cenocepacia Prophages—Prevalence, Chromosome Location and Major Genes Involved
Source: Viruses. 2018 May 31;10(6):297. doi: 10.3390/v10060297 (PMC6024312; doi:10.3390/v10060297)
Supplement: Supplementary file 1 [file viruses-10-00297-s001.zip › viruses-297954-r2-supplementary OK/Supplementary data/Region Characteristics Cards/Supplementary_data_9_RC_DWS 37E-2_chr1_1.docx]

| **Region characteristics** | | | |
| --- | --- | --- | --- |
| Phage name: | DWS 37E-2_chr1_1 | | |
| Size (nt): | 36859 | | |
| Type: | Prophage | | |
| Taxonomical affiliation (homology based): | Order: *Caudovirales*  Family: *Siphoviridae* | | |
| Number of annotated open reading frames (ORF): | 44 | | |
| Number of annotated regulatory sequences: | Terminators: | 13 | |
|  | Promoters: | 0 | |
|  | tRNA: | 0 | |
| Derivation: | Host: | | *Burkholderia cenocepacia* DWS 37E-2,  chromosome 1 |
|  | Sequence origin (database) | | NCBI |
|  | Accession number/version: | | NZ_CP007781.1 |
|  | Localization in genome: | | 2747954..2784813 |
|  | Additional information: | | - |
| Additional information: | - Phage poses sequences which may serve as *cos* sites  - Two integrases were identified - probably non-functional (too short in comparison to their homologues) (marked red)  - terminase gene is present - probably non-functional (too short in comparison to their homologues (marked red)  - complete lytic cassette was found in position 13839..15111  - in the closest neighborhood of the integrated genome *virulence protein E* (WP_040144422.1)  - trifling homology (2-4%) suggest derivation to *Siphoviridae*  - of the genes that were found in region:  a) 9 genes show homology with known phage genes  b) 16 genes are distinctive for phages, although with no homology to viral sequences in the database (green)  c) 19 genes with homology to bacterial genes (blue) | | |

| **Annotation** | | | | | |
| --- | --- | --- | --- | --- | --- |
| **#** | **Strand** | **Start** | **End** | **Length (nt)** | **Product** |
| - | x | 1 | 14 | 14 | attL |
| 1 | - | 3 | 182 | 180 | inactive integrase |
| 2 | - | 229 | 453 | 225 | inactive integrase |
| 3 | + | 637 | 1866 | 1230 | acyltransferase family protein |
| 4 | + | 3391 | 3891 | 501 | hypothetical protein |
| 5 | + | 3888 | 5012 | 1125 | hypothetical protein |
| 6 | + | 5239 | 7020 | 1782 | hypothetical protein |
| 7 | + | 7072 | 7635 | 564 | hypothetical protein |
| 8 | - | 7861 | 8790 | 930 | ATP-binding protein |
| 9 | - | 8804 | 9586 | 783 | hypothetical protein |
| 10 | + | 9585 | 10052 | 468 | hypothetical protein |
| 11 | - | 10311 | 10490 | 180 | hypothetical protein |
| 12 | - | 10919 | 12073 | 1155 | tad-like Flp pilus-assembly family protein |
| 13 | - | 12135 | 12557 | 423 | tadE-like family protein |
| 14 | - | 12880 | 13668 | 789 | DNA adenine methylase |
| 15 | - | 13839 | 14333 | 495 | Rz |
| 16 | - | 14330 | 14824 | 495 | endolysin |
| 17 | - | 14827 | 15111 | 285 | holin |
| 18 | - | 15186 | 16238 | 1053 | late control D protein |
| 19 | - | 16249 | 16455 | 207 | hypothetical protein |
| 20 | - | 16430 | 17311 | 882 | oxidoreductase |
| 21 | - | 17321 | 19738 | 2418 | tail tape measure protein |
| 22 | - | 19795 | 20124 | 330 | hypothetical protein |
| 23 | - | 20218 | 20721 | 504 | major tail tube protein |
| 24 | - | 20732 | 21901 | 1170 | tail sheath protein |
| 25 | - | 21977 | 22429 | 453 | tail protein |
| 26 | - | 22431 | 23549 | 1119 | hypothetical protein |
| 27 | - | 23546 | 24115 | 570 | tail protein |
| 28 | - | 24105 | 24998 | 894 | baseplate J protein |
| 29 | - | 24995 | 25330 | 336 | baseplate protein |
| 30 | - | 25330 | 25530 | 201 | hypothetical protein |
| 31 | - | 25599 | 26327 | 729 | hypothetical protein |
| 32 | - | 26440 | 27120 | 681 | baseplate protein |
| 33 | - | 27113 | 27643 | 531 | hypothetical protein |
| 34 | - | 27636 | 28163 | 528 | tail protein |
| 35 | - | 28169 | 28459 | 291 | hypothetical protein |
| 36 | - | 28461 | 29456 | 996 | minor capsid protein E |
| 37 | - | 29533 | 29877 | 345 | head decoration protein |
| 38 | - | 29902 | 31017 | 1116 | peptidase S14 |
| 39 | - | 31007 | 32494 | 1488 | portal protein |
| 40 | - | 32491 | 33138 | 648 | inactive terminase |
| 41 | - | 33165 | 34691 | 1527 | terminase large subunit |
| 42 | - | 34669 | 35223 | 555 | hypothetical protein |
| 43 | - | 35314 | 35508 | 195 | hypothetical protein |
| 44 | + | 35915 | 36289 | 375 | hypothetical protein |
| - | x | 36847 | 36860 | 14 | attR |

| **Terminators** | | | |
| --- | --- | --- | --- |
| **Strand** | **Start** | **End** | **Sequence** |
| - | 15136 | 15160 | GCCGCCTCGGGGCAACTCGGGCGGC |
| - | 20146 | 20167 | GGGGCGGTCCGTGGACCGCCCC |
| - | 20146 | 20167 | GGGCGGTCCGTGGACCGCCCC |
| - | 21932 | 21948 | GCCGCTCATTCAGCGGC |
| - | 25552 | 25570 | GCCCCGCCTAGTGCGGGGC |
| + | 25961 | 26983 | GGCTGGCGACATATTTAGCCAGC |
| - | 29490 | 29510 | GGCCACGCATCGCGCGTGGCC |
| + | 32763 | 32780 | CCCCGCTCGGTGGCGGGG |
| + | 33224 | 33240 | GGCCGTATTTCGCGGCC |
| - | 35264 | 35283 | TGCGGGGGCTCAGACCCGCG |
| - | 35529 | 35553 | GCCCCGGGTGCTAAAGCCTCGGGGC |
| - | 36366 | 36392 | GCCCTGAGTGCGGAACGCGCTCAGGGC |
| + | 36366 | 36392 | GCCCTGAGCGCGTTCCGCACTCAGGGC |
